# Supplementary material for: Phase II, double blind, placebo controlled, multi-site study to evaluate the safety, feasibility and desirability of conducting a phase III study of anamorelin for anorexia in people with small cell lung cancer: A study protocol (LUANA trial)
Source: PLoS One. 2023 May 17;18(5):e0285850. doi: 10.1371/journal.pone.0285850 (PMC10191352; doi:10.1371/journal.pone.0285850)
Supplement: S1 File — (PDF) [file pone.0285850.s002.pdf]

Protocol (Anamorelin for Anorexia – LUANA trial)

TITLE:	Phase II, double blind, placebo controlled, parallel arm, fixed dose, multi-site study to evaluate the safety, feasibility and desirability of conducting a fully powered phase III study of anamorelin for anorexia in people with small cell lung cancer.
PROTOCOL NUMBER	044/21 V1.6
STUDY MEDICINE	Anamorelin HCl, 100 mg daily, oral
CHIEF INVESTIGATOR	David Currow, FAHMS<br>Professor of Palliative Medicine<br>IMPACCT, Faculty of Health, University of Technology Sydney<br>PO Box 123, Ultimo, New South Wales, 2007, Australia<br>+61 2 9514 4862<br>david.currow@uts.edu.au
DATE OF PROTOCOL	07 April 2022
STUDY SPONSOR	University of Technology Sydney<br>15 Broadway, Ultimo NSW 2007, Australia<br>Email: ITCC@uts.edu.au
TRIAL REGISTRATION	ACTRN12622000129785 (27 January 2022)

**Investigator Team**			
**Name**	**Faculty/Department**	**Telephone**	**Qualifications**
**Coordinating Principal Investigator**			
**Prof David Currow**	IMPACCT, University of Technology Sydney, NSW, Australia	02 9514 4862	BMed, MPH, PhD, FRACP, FAHMS
**Principal Investigators**			
**Prof Peter Martin**	Deakin University, Geelong, Victoria, Australia	03 4215 5105	MB, BCh, BAO, PGDipPM, MMed, FAChPM
**Prof Miriam Johnson**	Wolfson Palliative Care Research Centre, University of Hull, Hull, England. HU6 7RX	+44 01482 46 3309	MD, FRCP, MRCP, MBChB(hons)

Investigator Team			
**Name**	**Faculty/Department**	**Telephone**	**Qualifications**
**Dr Victoria Bray**	Department of Medical Oncology, Liverpool Hospital, NSW, Australia	02 8738 9736	MBBS, FRACP, PhD
**Dr Matthew Maddocks**	King's College London, Denmark Hill, London UK	+44 020 7848 5242	FHEA, PhD, BSc(Hons) MCSP
**Ms Alex Bullock**	Wolfson Palliative Care Research Centre, Hull York Medical School, University of Hull, Hull, England. HU6 7RX	+44 01482 462217	RD, MNutr
**Dr Mariana Sousa**	IMPACCT, University of Technology Sydney, NSW, Australia	0404357571	MD, PhD
**Dr Slavica Kochovska**	IMPACCT, University of Technology Sydney, NSW, Australia	02 9514 3978	BA(Hons), MA(Hons 1), PhD
**Dr Sungwon Chang**	IMPACCT, University of Technology Sydney, NSW, Australia	02 9514 4655	BSc, MStat, PhD
**Dr Deidre Morgan**	RePaDD, Flinders University, Adelaide, SA, Australia	08 72218220	BAppSc (OT), MCSc (OT), PhD
**Prof Meera Agar**	IMPACCT, University of Technology Sydney, NSW, Australia	0430 212 912	MBBS (Hons 1) MPC, FRACP FAChPM PhD
**Prof Michael Lind**	Hull University Teaching Hospital Trust, Hull England	+44 01482 461260	MBBS, FRCP
**Dr Irina Kinchin**	Trinity College Dublin, the University of Dublin, Dublin	+353 1 896 1000	PhD, MSc
**Consumer Representative**			
**Mr John Stubbs**	IMPACCT, University of Technology Sydney, NSW, Australia		BA, Dip Acct.
**Dr Phillip Lee**	IMPACCT, University of Technology Sydney, NSW, Australia		MBBS, PGDip Pall Med, FAChPM
**Associate Investigators**			
**Ms Belinda Fazekas**	IMPACCT, University of Technology Sydney, NSW, Australia	0414 190 084	BN, GradDipComm Health

Investigator Team			
**Name**	**Faculty/Department**	**Telephone**	**Qualifications**
**Ms Linda Brown**	IMPACCT, University of Technology Sydney, NSW, Australia	02 9514 4862	MBus, BBus (Management)
**Dr Valentina Naumovski**	IMPACCT, University of Technology Sydney, NSW, Australia	0403113182	BE (Hons), BSc (CHEM), PhD, GCULT
**Study Statistician**			
**Dr Sungwon Chang**	IMPACCT, University of Technology Sydney, NSW, Australia	02 9514 4655	BSc, MStat, PhD
**Health Economist**			
**Dr Irina Kinchin**	Trinity College Dublin, the University of Dublin, Dublin	+353 1 896 1000	PhD, MSc

Regulatory Statement

All study procedures will be conducted within ICH GCP guidelines (TGA annotated version) and all other regulatory requirements.

Protocol Preparation

This protocol has been built with the guidance and consideration of the following:

- ICH GCP R2 Section 6, Clinical Trial Protocol
- Quality in clinical trials (CTTI) aims to reduce errors that matter i.e. Errors that impact on participant's safety and the reliability of data. Critical quality points to consider are listed as CTTI factors
- Australian Privacy Principles (APP) guidelines [available at https://www.oaic.gov.au/privacy/australian-privacy-principles-guidelines/]
- Standard Protocol Items: Recommendations for Interventional Trials (SPIRIT): https://www.spirit-statement.org/
- SPIRIT-Pro Extension: http://www.pocog.org.au/doc/SPIRIT-PRO_Checklist_for-CTG-use_v0.2.pdf
- CONSORT. International Committee of Medical Journal Editors. Uniform requirements for manuscripts submitted to biomedical journals. Annals of Internal Medicine. 1997. 126; 36-47.

**Protocol History**			
Version	Date	Author	Reason
1.1	11/04/2021	David Currow	New protocol from draft
1.2	08/07/2021	David Currow	Finalised version
1.3	25/08/2021	David Currow	HREA submission version
1.4	26/10/2021	David Currow	Amendment post HREC review
1.5	27/01/2022	David Currow	Change in third party pharmacy details

**Protocol Approval**			
Version	Date	Approval Name	Approval Signature
1.3	8/07/2021	David Currow	Email approval
1.4	10/11/2021	David Currow	Email approval
1.5	27/01/2022	David Currow	Email approval
1.6	07/04/2022	David Currow	Email approval

TRIAL SUMMARY

Background

Up to 57% of people with small cell lung cancer (SCLC) present with considerable unintentional weight loss at the time of diagnosis and with disease progression the prevalence of anorexia is as high as 66% in this group. These changes are associated with worsening of functional capacity and tolerance to treatment, leading to poorer quality of life, prognoses, and outcomes. Despite the significant importance of cancer-related anorexia, treatments are lacking and there are no medications approved for this indication.

Anamorelin, by virtue of its ghrelin receptor agonist activity (i.e. anabolic and appetite-enhancing), may serve a role in the treatment of cancer-related anorexia. Clinical trials have shown the drug to be safe and effective in improving lean body mass and body weight in patients with non-small cell lung cancer (NSCLC). However, these trials failed to meet functional co-primary endpoint while at the same time showing that the medication was well tolerated and sustainably improved appetite in the majority of participants, a finding not seen in the placebo control arm.

Considering the unmet clinical need for safe and effective treatments for anorexia and the existing clinical trial data, anamorelin might improve anorexia and, at the same time, increase tolerance to anti-cancer therapies and maintenance of function, thus improving quality of life for people with SCLC and other cancers.

We propose to conduct a Phase II trial to determine the safety, feasibility and desirability of a fully powered multi-site, double-blind, randomized, phase III trial of anamorelin HCl (100 mg) /placebo for treating anorexia in people with SCLC. The trial design includes optimal measurable endpoints that will allow the most informative and clinically significant data to be obtained and inform the design of a robust effectiveness trial. In addition, a mixed-methods sub-study will help address patients' and caregivers' unmet needs as it will improve understanding of the conversations that occur during patient-clinician consultations regarding clinical history taking of cancer-related symptoms and their impacts.

Study design

This is a phase II, randomised, double-blind, placebo controlled, parallel arm, fixed dose, multi-site study.

Target Study Population

Adults (aged ≥18 years) newly diagnosed with small cell lung cancer with planned systemic therapy OR with first recurrence of disease following successful treatment with a documented disease-free interval of at least ≥ 6 months, AND with anorexia (i.e. ≤ 37 points on the 12-item Functional Assessment of Anorexia Cachexia Treatment (FAACT A/CS) scale).

Objectives

Primary: To demonstrate safety, desirability (sufficient signal in the efficacy evaluation) and feasibility of conducting a fully powered phase III study of anamorelin HCl (100mg) for the treatment of anorexia in people with SCLC. Primary efficacy parameters will include:

- Change in anorexia-related symptoms and concerns;
- rates of *on time, on dose* completion of any administered chemotherapy and/or radiotherapy.

Secondary: Secondary efficacy parameters will include:

- change in body weight;
- change in lean body mass and muscle mass at thoracic (T4) level;
- functional status;
- performance-based functional mobility and physical activity;
- nutritional intake;
- biochemical changes;
- survival (overall and cancer-specific);
- participant's and clinician's rating of overall improvement;
- quality of life;
- chronic illness therapy-fatigue;
- rates of unplanned health care contact (including inpatient/outpatient attendance and community care such as emergency department contact, unplanned primary care or specialist contact, etc.);
- rates of hospitalisations and length of stay;
- adverse events from chemotherapy (e.g. nausea, vomiting, anorexia, fatigue);
- rates of febrile neutropaenia;
- harms.

Tertiary: To assess:

- caregivers' unmet needs and quality of life;
- participants' and caregivers' indirect and informal care costs and resource use.

In addition, a mixed-methods sub-study will run in parallel with the main trial to ascertain:

- patients', caregivers' and clinicians' concerns around the impact of cancer-related symptoms on patient's quality of life; and
- whether and how these issues are discussed in clinical consultations (if at all).

The sub-study will be detailed in a separate document – the Sub-study Procedures Manual.

Intervention

Group 1 / intervention arm – oral anamorelin HCl 100 mg once daily (administered as tablets in the fasted condition – ≥1 hour before the first meal of the day).

Group 2 / control arm – placebo once daily (administered as matching placebo tablets regarding appearance, smell and taste, in the fasted condition – ≥1 hour before the first meal of the day).

Study procedures

Participants will be recruited using advertisement in waiting rooms and clinician referrals. Adults (aged ≥18 years) with a confirmed diagnosis of SCLC are referred to the study nurse at each participating centre and assessed for eligibility. People who meet **all** of the inclusion criteria and **none** of the exclusion criteria are eligible to enter this study. All participants will be required to sign an informed consent prior to performing any study procedures. Screening assessments will be undertaken to confirm eligibility. Randomisation will occur after eligibility is confirmed and baseline assessments are collected. A total of 50 participants with SCLC and anorexia will be randomised 1:1 to oral anamorelin HCl 100mg (Group 1 / intervention arm) or placebo (Group 2 / control arm) once daily (≥1 hour before first meal of the day) for at least 12 weeks. Treatment (Day 1) will initiate as soon as practicable; ideally prior to cycle 1 of chemotherapy and **no later** than one day prior to scheduled date of cycle 2 of planned chemotherapy. If chemotherapy is not planned, Day 1 should occur within three weeks of screening visit. All participants will receive standard physical activity and diet advice leaflets at baseline from the study nurse as part of a multi-modal intervention. At the end of the 12 weeks, participants are invited to enrol in an extension phase where they will continue to receive blinded intervention medication for another 12 weeks. The optional extension (weeks 13-24) is entirely based on participant preference. Participants will visit the study centre at baseline, weeks 4, 8 and 12 (plus 18 and 24 for those in the extension phase). During the visits, the

study nurse will take the measures and assessments outlined below. Primary and secondary efficacy analysis will be conducted at 12 weeks. Additional exploratory efficacy and safety analyses will also be conducted at 24 weeks (for those who opt in to the extension phase) in order to collect data over longer treatment duration.

Study Diagram

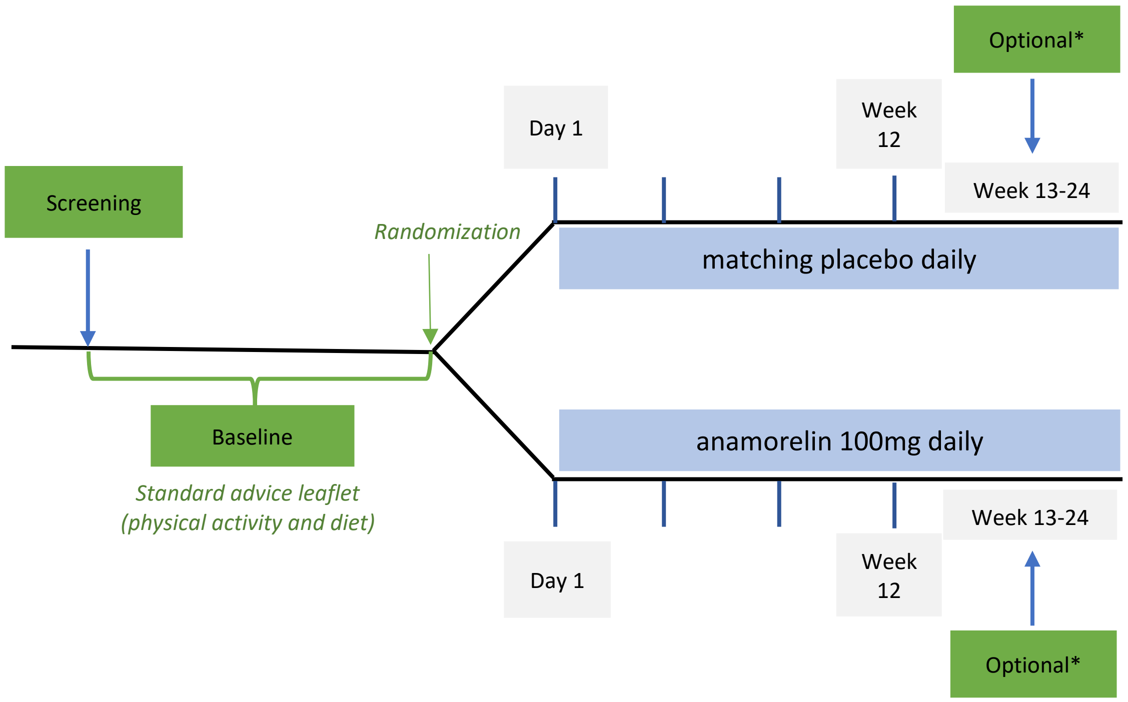

**The optional extension is entirely based on participants' preference for blinded continuation for weeks 13-24.*

Endpoints and assessments

Primary - Feasibility

Progression criteria to a Phase III study will be assessed using an indicative traffic light system (green, orange red) whereby green (go) suggests that the criteria have been met and the trial should proceed, orange (amend) indicates that some changes should be made to the larger trial and red (stop) indicates that a larger trial should not proceed. Feasibility in this trial is related to:

- recruitment rate, defined as an average of one participant per site (six sites) every six weeks – plan to recruit 50 participants;
- proportion of participants with complete primary endpoint data – >70% (green), 60-70% (orange), <60% (red);

- participant adherence to investigational medication – >70% (green), 60-70% (orange), <60% (red);
- participant adherence to physical activity advice, as measured by the Short Questionnaire to Assess Health Enhancing Physical Activity (SQUASH), completed at every visit (whereby increase in physical activity will be reviewed over the study period – >40% (green), 30-40% (orange), <30% (red);
- participant adherence to dietary advice, as measured by the food diary kept for three days prior to visits (whereby increase in macronutrient intake will be reviewed over the study period – >40% (green), 30-40% (orange), <30% (red).

***Primary - Desirability*:** The study will be considered desirable if there is sufficient signal in the efficacy evaluation to suggest that an adequately powered study would deliver a clinically meaningful difference between groups (where the primary measure has a minimum clinically important difference pre-defined), feasibility endpoints are met and participants find the study acceptable.

Primary - Efficacy for clinical assessment

- Change in the 5-item Anorexia Symptom Scale Domain from the 12-item Functional Assessment of Anorexia Cachexia Treatment (FAACT, anorexia/cachexia – A/CS) scale by arm from baseline to week 12.
- Rates of *on time, on dose* completion of any administered chemotherapy and/or radiotherapy.

Secondary - Clinical assessment (proposed for any subsequent phase III study)

- Baseline demographic and clinical predictors of responders.
- Body weight, BMI and historical stable weight.
- Lean body mass (measured by change in dual x-ray absorptiometry (DEXA) and Bioelectrical Impedance Analysis – BIA) and muscle mass at T4 (measured by computed tomography -CT scan) by arm.
- Functional status using clinician-rated Australia-modified Karnofsky Performance Scale (AKPS).
- Timed up and go test (TUG).
- Dynamic function as measured by a pedometer app or a wearable digital pedometer, for participants who do not have mobiles, worn for two days prior to visits.
- Nutritional intake measured by self-reported 3-day food diary, including 2 weekdays and one weekend day prior to visits. If participants do not complete the 3-day food diary prior to visits, a 24-h diet recall will be collected by the study nurse at the time of visit.
- Biochemical changes (including IL-6, CRP, albumin and pre-albumin).

- Survival (overall and cancer-specific).
- Participant rated global impression of change (GIC).
- Clinician rated clinical global impression of severity and improvement (CGI-S, CGI-i)
- Quality of life assessed with EQ-5D-5L – EuroQoL five-dimensional instrument, and ICECAP-A – ICEpop CAPability measure for Adults.
- Participant-reported fatigue as measured by the Functional Assessment of Chronic Illness Therapy-Fatigue (FACIT-F) domain.
- Unplanned health care contacts including hospitalisations.
- Episodes of febrile neutropaenia.
- Adverse events from chemotherapy (e.g., nausea, vomiting, anorexia, fatigue).
- Harms in each arm.

Tertiary

- Caregivers' quality of life (EQ-5D-5L – EuroQoL five-dimensional instrument), and unmet needs (Carer Experience Scale – CES).
- Capture participants and caregivers indirect and informal care costs and resource use (e.g. time off work, time spend in-home nursing or accompanying to hospital, any 'out of pocket' expenses).

Statistical analysis

Sample Size

Since this is a feasibility study, it is not required to calculate a fully justified sample size for establishing the efficacy of the intervention.[1] It has been suggested, however, that in feasibility studies, a sample size between 24 and 50 is acceptable.[2,3] The findings from this study will be used to inform the calculation of a sample size for a future larger scale phase III trial.

---

[1]Arain M, Campbell MJ, Cooper CL, Lancaster GA. What is a pilot or feasibility study? A review of current practice and editorial policy. BMC Med Res Methodol. 2010;10:67).
[2] Sim J, Lewis M. The size of a pilot study for a clinical trial should be calculated in relation to considerations of precision and efficiency. J Clin Epidemiol. 2012;65(3):301–8.
[3]Julious SA. Sample size of 12 per group rule of thumb for a pilot study. Pharm Stat 2005;4:287-291

Primary analysis will be concentrated on the feasibility metrics based on defined thresholds. As this is a feasibility trial, there will be no imputation. Data relating to quantitative variables will be analysed with simple frequencies. Comparison between the intervention and control groups will be compared using appropriate statistical approaches, depending on the nature of the data.

Efficacy outcomes: Changes from baseline to 12 weeks or 24 weeks in each of the secondary endpoints will be calculated. An exploratory analysis of the potential differences between arms will be performed using t-test for continuous normally distributed data or Wilcoxon Test for continuous non-normally distributed data.

Safety outcomes**:** All safety and tolerability data will be summarised descriptively. Predictors of harms will be sought in order to inform refinement of the phase III study eligibility. Descriptive statistics will be produced for each of the outcome measures to evaluate the appropriateness of the measures for inclusion in a definitive trial.

Economic analysis: The feasibility of collecting data for a comprehensive cost effectiveness analysis of anamorelin in people with SCLC and anorexia will be determined in any subsequent phase III clinical trial.

CONTENTS

PROTOCOL (ANAMORELIN FOR ANOREXIA – LUANA TRIAL) **1**

CONTENTS **12**

1.0 BACKGROUND AND RATIONALE **18**

1.1 REVIEW OF THE LITERATURE 18
1.2 RATIONALE FOR STUDY POPULATION 18
1.3 EXISTING EVIDENCE OF ANAMORELIN FOR ANOREXIA 19
1.4 RATIONALE FOR SELECTED DOSE RANGE 20
1.5 RELEVANT SAFETY AND TOXICITY INFORMATION 20
1.6 RATIONALE FOR STUDY PROTOCOL AND DESIGN 20

2.0 STUDY HYPOTHESIS, OBJECTIVES AND PURPOSE **22**

2.1 RESEARCH HYPOTHESIS 22
2.1.1 Alternative Hypotheses (H1) *22*
2.2 OBJECTIVES 22
2.2.1 Primary objective *22*
2.2.2 Secondary objectives *22*
2.2.3 Tertiary objectives *23*

3.0 STUDY DESIGN, SETTING AND POPULATION **24**

3.1 STUDY DESIGN 24
3.2 STUDY DIAGRAM 25
3.3 STUDY SETTING 25
3.4 STUDY POPULATION 25
3.4.1 Target population *25*
3.4.2 Eligibility criteria *25*
3.4.3 Withdrawal criteria *29*

4.0 INTERVENTION **30**

4.1 STUDY MEDICATION 30
4.2 DOSING SCHEDULE 31
4.3 METHOD OF ASSIGNING PARTICIPANTS TO TREATMENT GROUPS 32
4.4 BLINDING 32
4.5 UNBLINDING PROCEDURES 33
4.6 DRUG SUPPLY 33
4.7 DRUG ACCOUNTABILITY AND DESTRUCTION 34
4.8 CESSATION OF STUDY DRUG/INTERVENTION 34
4.9 TREATMENT FAILURE 35
4.10 STOPPING RULES 36

5.0 OUTCOMES AND MEASURES **37**

5.1 PRIMARY OUTCOME 37
5.1.1 Feasibility *37*
5.1.2 Desirability *37*
5.1.3 Efficacy (for clinical assessments) *38*
5.2 SECONDARY ENDPOINTS 38
5.2.1 Secondary efficacy (clinical assessments proposed for any subsequent phase III study) *38*
5.2.2 Safety Assessments *39*
5.3 TERTIARY ENDPOINTS 39
5.3.1 Cost effectiveness and caregivers' assessments *39*

6.0 STUDY ASSESSMENTS ... 41

6.1 Laboratory measures ... 41
6.2 Clinical and physical measurements ... 42
6.3 Comorbidity assessment ... 45
6.4 Demographics ... 45
6.5 Main clinical diagnosis ... 46
6.6 Functional status ... 46
6.7 Patient reported symptom measure ... 47
6.8 Caregiver reported measure ... 50
6.9 Participant retention and follow-up ... 50

7.0 PARTICIPANT TIMELINE ... 55

7.1 Visit 1 (Screening) ... 55
7.2 Visit 2 (Baseline/ Randomisation) ... 55
7.3 Visit 3 (Week 4) ... 57
7.4 Visit 4 (Week 8) ... 57
7.5 Visit 5 (Week 12) – PRIMARY ENDPOINT ... 58
7.6 Visit 6 (Optional Extension Phase - Week 18) ... 59
7.7 Visit 7 (Optional Extension Phase - Week 24) ... 59
7.8 Study visit schedule diagram ... 61

8.0 STUDY PROCEDURE ... 62

8.1 Referrals ... 62
8.2 Participant Informed consent process ... 62
8.3 Caregiver consent ... 64
8.4 Screening for eligibility ... 64
8.5 Re-screening ... 65
8.6 Procedure to request randomisation ... 66
8.7 Prescription of study drugs ... 66
8.8 Treatment commencement ... 67
8.9 Assessment visits ... 67
8.10 End of treatment assessments ... 67
8.11 Withdrawal assessments ... 68
8.12 Follow-up phase assessments ... 68
8.13 Extension phase criteria ... 69
8.14 Sub-study ... 69

9.0 STUDY ADMINISTRATION AND MONITORING ... 70

9.1 Reporting of adverse events ... 70
9.1.1 The Trial Management Committee ... 70
9.1.2 Human Research Ethics Committees ... 70
9.1.3 Medical Monitor ... 70
9.1.4 Adverse events ... 71
9.1.5 Serious adverse events ... 71
9.1.6 Criteria for assessing severity ... 73
9.1.7 Criteria for assessing causality ... 73
9.1.8 Time period for assessing AEs and SAEs ... 74
9.1.9 National Cancer Institute, Common Terminology Criteria for Adverse Events. (NCI CTC AE).V5.0 ... 75
9.1.10 Adverse Event Assessment Diagram ... 76
9.1.11 Follow-up of AEs and SAEs ... 77
9.1.12 Post study AEs and SAEs ... 77
9.2 Auditing/Monitoring ... 77
9.2.1 Peer review and site visits ... 77

9.2.2 Pharmacy training ... *78*
9.2.3 Monitoring visits ... *78*
9.3 QUALITY CONTROL ... 78
9.3.1 Training procedures: ... *78*
9.3.2 Blood collection ... *79*
9.4 DATA MANAGEMENT ... 80
9.4.1 Data handling and record keeping ... *80*
9.4.2 Direct access to source data ... *80*
9.4.3 Data collection ... *81*
9.4.4 Electronic recording ... *82*
9.4.5 Data entry ... *82*
9.4.6 Data querying ... *83*
9.4.7 Data storage ... *83*
10.0 STATISTICS ... 84
10.1 POWER AND SAMPLE SIZE ... 84
10.2 ALTERNATIVE HYPOTHESES TO BE TESTED ... 84
10.3 ANALYSIS OF PRIMARY OUTCOMES ... 84
10.4 SAFETY OUTCOMES ... 85
10.5 ECONOMIC ANALYSIS ... 85
11.0 ETHICS AND DISSEMINATION ... 86
11.1 BENEFIT ANTICIPATED FROM THE STUDY ... 86
11.2 ASSESSMENT OF BURDEN AND STRESS ... 86
11.3 POTENTIAL RISKS ... 87
11.3.1 Participants of non-English speaking background ... *87*
11.3.2 Non-participation and withdrawal from the study ... *88*
11.3.3 Use of an unapproved medicinal product/novel intervention: ... *88*
11.3.4 Risks associated with dose administration ... *88*
11.3.5 Risks associated with study procedures- DEXA scans ... *89*
11.4 CONFIDENTIALITY ... 89
11.4.1 Separation of research and clinical responsibilities ... *89*
11.4.2 Protection of privacy and preservation of confidentiality ... *90*
11.4.3 Use of personal information ... *90*
11.4.4 Retention and Disposal of personal information ... *90*
11.5 METHOD AND NATURE OF RECRUITMENT AND ADVERTISING ... 91
11.6 ACCESS TO DATA ... 91
11.7 SPONSOR AND COLLABORATIONS ... 91
11.8 DISSEMINATION POLICY ... 92
11.9 AUTHORSHIP ... 92
12.0 REFERENCES ... 93
13.0 AMENDMENT LISTING ... 96

LIST OF ABBREVIATIONS

µg	*microgram*
A/CS	*12-item Additional Concerns Subscale of the FAACT*
AE	*Adverse Event*
AI	*Associate investigator*
AIDS	*Acquired immunodeficiency syndrome*
AKPS	*Australia – modified Karnofsky Performance Status*
ALP	*Alkaline phosphatase*
ALT	*Alanine transaminase (SGPT)*
AST	*Aspartate transaminase (SGOT)*
A-V block	*Atrioventricular block or Heart block*
BIA	*Biolectrical Impedance Analysis*
BMI	*Body mass index*
CBC	*Complete Blood Count*
CES	*Carer Experience Scale*
cm	*Centimetres*
CRF	*Case Report Forms*
CRP	*C-Reactive Protein*
CST	*Cancer Symptom Trials*
CT	*Computed Tomography*
CTN	*Clinical Trial Notification*
CYP3A4	*Cytochrome P450 3A4*
DEXA	*dual x-ray absorptiometry*
ECG	*Electrocardiogram*
eCRF	*Electronic Case Report Form*
EQ-5D-5L	*EuroQoL five-dimensional instrument*
FAACT	*Functional Assessment of Anorexia/Cachexia Treatment*
FACIT-F	*Functional Assessment of Chronic Illness Therapy-Fatigue*
FDA	*Food and Drug Administration*
GCP	*Good Clinical Practice*

GH	*Growth hormone*
GHSR-1a	*Growth hormone secretagogue receptor-1a*
GI	*Gastrointestinal*
GP	*General practitioner*
H0	*Null hypothesis*
H1	*Alternative hypothesis*
HbA1c	*Hemoglobin A1c*
HREC	*Human Research Ethics Committee*
ICECAP-A	*ICEpop CAPability measure for Adults*
ICH GCP	*International Conference on Harmonisation, Good Clinical Practice*
ID	*Identification number*
IMPACCT	*Improving Palliative, Aged and Chronic Care through Clinical Research and Translation*
ITCC	*IMPACCT Trials Coordination Centre*
kg	*Kilogram*
mg	*milligram*
mL	*millilitre*
MM	*Medical Monitor*
mmHg	*Millimetres of mercury*
MDRD	*Modification of Diet in Renal Disease*
NCI-CTCAE	*National Cancer Institute Common Criteria for Adverse Events*
NSCLC	*Non-small cell lung cancer*
NYHA	*New York Health Association*
PaCCSC	*Palliative Care Clinical Studies Collaborative*
PBS	*Pharmaceutical Benefits Scheme*
PE	*Physical examination*
PGIC	*Patient Global Impression of Change*
PGIS	*Patient Global Impression of Severity*
PI	*Principal Investigator. The investigator who leads the study conduct at an individual study centre. Every study centre has a principal investigator.*
PICF	*Participant Information and Consent Form*
PMI	*Participant Master Index*

PR interval	*PR interval is the time from the beginning of the P wave (atrial depolarization) to the beginning of the QRS complex (ventricular depolarization)*
PRO	*Patient Reported Outcomes*
QOL	*Quality of life*
QRS interval	*The QRS interval represents the electrical impulse as it spreads through the ventricles and indicates ventricular depolarization*
QT Interval	*The relationship between two conduction points on an electrocardiograph (ECG)*
RECIST	*Response evaluation criteria in solid tumors*
RGO	*Research Governance Office*
SAE	*Serious Adverse Event*
SBT	*Short Blessed Test*
SCLC	*Small cell lung caner*
SOP	*Standard Operating Procedure*
SQUASH	*Short Questionnaire to Assess Health Enhancing Physical Activity*
TGA	*Therapeutic Goods Administration*
TMC	*Trial Management Committee*
TUG	*Timed up and go test*
ULN	*Upper limit of normal*
WBC	*White blood cells*
WPW	*Wolff-Parkinson-White syndrome*

1.0 BACKGROUND AND RATIONALE

1.1 Review of the literature

Cancer-related anorexia (appetite loss) remains one of the most prevalent and bothersome clinical problems experienced by people with cancer during and after therapy.[1] It is estimated that over 50% of people present with anorexia at the time of a cancer diagnosis[1] and that it is an independent negative prognostic factor for survival.[2]

Cancer anorexia is a major contributor to weight loss and adversely influences nutritional status in advanced cancer.[1] People with anorexia generally have reduced physical capacity, often reporting being too weak to perform daily tasks, thus losing independence and time with their loved ones.[3] Anorexia also reduces the effectiveness of chemotherapy and the ability of people to cope with and complete their treatment (on-time and on-dose).[4] Cancer anorexia has also been linked to lower social functioning and depression, with profound impacts on the individual's psychological wellbeing and social life, all of which adversely affect quality of life (QoL).[1] Results from a cross-sectional survey assessing the burden and concerns caused by cancer-related weight loss on the lives of people with lung cancer, indicated that those with weight loss reported significantly lower overall QoL (55% vs 67%), and higher symptomatology: fatigue (65% vs 49%), nausea (20% vs 9%), and anorexia (41% vs 24%) than patients without weight loss. In addition, significantly more people who lost weight reported moderate/high distress levels than those who did not (71% vs 38%).[5]

In summary, anorexia, along with associated weight loss are common, debilitating, and concerning occurrences for people with cancer.[1] Despite the significant impact of cancer-related anorexia, treatments are lacking and there are no medications approved for this indication. Therefore, the treatment of malignancy-associated anorexia in people with cancer remains an area of unmet medical need.

1.2 Rationale for study population

Lung cancer is one of the most commonly occurring cancers in Australia and worldwide, representing over 12% of the global cancer burden and continues to be the leading cause of cancer-related deaths.[6] There are two main sub-types of lung cancer: non-small cell lung cancer (NSCLC) and small cell lung cancer (SCLC), with the latter form associated with greater mortality. People with SCLC have very high prevalence of weight and appetite loss,[7] and therefore, are being chosen for investigation in this study. It has been reported that up to 57% of people with SCLC present with considerable unintentional weight loss at the time of diagnosis[8] and with disease

progression the prevalence of anorexia is as high as 66% in this group.[9, 10] These changes are associated with worsening of functional capacity and tolerance to treatment, leading to poorer prognoses and outcomes.[11] As this study is investigating improvements in anorexia symptoms, people enrolled must demonstrate ongoing problems with their appetite/eating.

1.3 Existing evidence of anamorelin for anorexia

Ghrelin is an endogenous stimulator of appetite by targeting the growth hormone secretagogue receptor (GHSR-1a). Similar to ghrelin, anamorelin increases GH, insulin-like growth factor-1, and insulin-like growth factor binding protein-3 thus producing the desired effect of significant appetite stimulation. Two international, phase III trials (ROMANA 1 [HT-ANAM-301] and ROMANA 2 [HT-ANAM-302])[12, 13] and an associated extension study (ROMANA 3 [HT-ANAM-303])[14] tested anamorelin for cancer anorexia and cachexia in people with stage III and IV NSCLC. These placebo-controlled trials, which included a total of over 900 patients, demonstrated that anamorelin safely reversed muscle loss, augmented body weight and was associated with clear improvements in appetite. The trials failed to meet functional co-primary endpoint (in this case, hand-grip strength, which was a requirement for approval in Europe and the USA) but showed that anamorelin was well tolerated and sustainably improved appetite in the majority of participants, a finding not seen in the placebo control arm. That said, in these trials food intake was not recorded and it is not known if the improvement in anorexia translated into an adequate nutritional intake.[15] Therefore, despite promising findings, anamorelin has not been approved by the Therapeutic Goods Administration (TGA) in Australia or the Food and Drug Administration (FDA) in the US for use in cancer anorexia. The main regulatory concern has been the lack of reliable and clinically meaningful endpoints in these trials to show relevant effect on patient functioning and quality of life. By contrast, this proposed feasibility study uses a combination of participant-reported measures (appetite) and objective measures directly related to potentially improved health outcomes (improved ability to tolerate and complete anti-cancer therapies on-time and on-dose). These end-points relate directly in the proposed study to outcomes that can deliver improved overall health and wellbeing. Recently, the use of anamorelin for improvement of weight loss and anorexia in cancer cachexia in people with NSCLC, gastric cancer, pancreatic cancer and colorectal cancer was approved in Japan, a world first.[16] Consequently, there is considerable expected value from the current proposed trial which includes endpoints relevant to completing disease-modifying cancer therapy and better maintaining functional status.

1.4 Rationale for selected dose range

In this study, 100 mg was selected as the dose of anamorelin HCl. This dose had been evaluated in previously conducted Phase III studies (HT-ANAM-301 and HT-ANAM-302)[12, 13] in NSCLC patients indicating that it was well-tolerated and showed improvement in cancer anorexia symptoms/concerns in people with NSCLC. A positive anabolic effect through both increased body weight and lean mass was also observed. In the safety extension study (HT-ANAM-303),[14] where participants from Studies HT-ANAM-301/302 continued dosing for up to an additional 12 weeks, a 100 mg dose level was also well tolerated, with no new safety signals identified in the longer dosing period. Due to the lack of safe and effective treatments for anorexia in people with cancer, placebo treatment will be used in the control group in this study.

1.5 Relevant safety and toxicity information

The current safety profile of anamorelin was established from the ROMANA trials.[12, 14] The most common drug-related treatment emergent harms observed were diabetes and hyperglycaemia, occurring in <1% of patients in ROMANA1 and ROMANA 2 trials. Anamorelin also induced prolongation of PR and QRS intervals at supratherapeutic doses of 300 and 400mg (based on 24-hour Holter monitoring data from Study HT-ANAM-112).[12, 14] The electrocardiogram (ECG) effects were then reassessed in a thorough QT/QTc study (Study HT-ANAM-113)[13, 14] which confirmed that anamorelin had no meaningful effect on ventricular repolarization. All ECG changes were transient, and none of the QT prolongations were above an absolute value of 450ms or were an increase of >60ms from baseline. Other associated harms were nausea, diarrhea, peripheral oedema and fatigue. In view of this, this study will specifically seek evidence of cardiac dysfunction, symptomatic hyperglycaemia, drug interactions, constipation, nausea and vomiting using National Cancer Institute Common Terminology Criteria for Adverse Event (NCI-CTCAE) gradings. Importantly, this proposed trial is using a dose of 100mg which has not been clinically associated with cardiac dysfunction.

1.6 Rationale for study protocol and design

This study is proposing to target anorexia in people with SCLC, as this symptom is rated amongst the top concerns for them (second only to fatigue) and there are currently limited treatment options.[1] Considering the unmet clinical need for safe and effective treatments for anorexia, anamorelin might improve anorexia and, at the same time, increase tolerance to anti-cancer therapies and maintenance of function, thus improving quality of life for people with SCLC and other cancers. We propose to conduct a Phase II trial to determine the safety, feasibility

and desirability of a fully powered multi-site, double-blind, randomized, phase III trial of anamorelin HCl (100 mg) /placebo for treating anorexia in people with SCLC. The trial design includes optimal measurable endpoints that will allow the most informative and clinically significant data to be obtained and inform the design of a robust effectiveness trial. In addition, a mixed-methods sub-study will help address patients' and caregivers' unmet needs as it will improve understanding of the conversations that occur during patient-clinician consultations regarding clinical history taking of cancer-related symptoms and their impacts.

2.0 STUDY HYPOTHESIS, OBJECTIVES AND PURPOSE

2.1 Research Hypothesis

2.1.1 Alternative Hypotheses (H1)

- Feasibility: There will be adequate recruitment and retention of participants with SCLC and anorexia for the phase II trial.

- Desirability: Anamorelin will show sufficient clinical signal of difference between the treatment and control arms with acceptable safety measures.

2.2 Objectives

2.2.1 Primary objective

To demonstrate safety, desirability (sufficient signal in the efficacy evaluation) and feasibility of conducting a fully powered phase III study of anamorelin HCL (100mg) for the treatment of anorexia in people with SCLC. Primary efficacy parameters will include:

- change in anorexia-related symptoms and concerns;
- rates of *on time, on dose* completion of any administered chemotherapy and/or radiotherapy.

2.2.2 Secondary objectives

Secondary efficacy parameters will include:

- change in body weight;
- change in lean body mass and muscle mass at thoracic (T4) level;
- functional status;
- performance-based functional mobility and physical activity;
- nutritional intake;
- biochemical changes;
- survival (overall and cancer-specific);
- participant's and clinician's rating of overall improvement;
- quality of life;

- chronic illness therapy-fatigue;
- rates of unplanned health care contact (including inpatient/outpatient attendance and community care such as emergency department contact, unplanned primary care or specialist contact, etc.);
- rates of hospitalisations and length of stay;
- adverse events from chemotherapy (e.g. nausea, vomiting, anorexia, fatigue);
- rates of febrile neutropaenia;
- harms.

2.2.3 Tertiary objectives

To assess:

- caregivers' unmet needs and quality of life;
- participants' and caregivers' indirect and informal care costs and resource use.

In addition, a mixed-methods sub-study will run in parallel with the main trial to ascertain:

- patients', caregivers' and clinicians' concerns around the impact of cancer-related symptoms on patient's daily life; and
- whether and how these issues are discussed in clinical consultations (if at all).

The sub-study will be detailed in a separate document – the Sub-study Procedures Manual.

3.0 STUDY DESIGN, SETTING AND POPULATION

3.1 Study design

This is a multi-site, randomised, double-blind, placebo-controlled, parallel arm, fixed dose, phase II study.

A total of 50 participants with SCLC and anorexia will be randomised 1:1 to oral anamorelin HCl 100mg (Group 1 / intervention arm) or placebo (Group 2 / control arm) once daily (≥1 hour before first meal of the day) for at least 12 weeks. Study treatment (Day 1) will start as soon as practicable; ideally prior to cycle 1 of chemotherapy and **no later** than one day prior to scheduled date of cycle 2 of planned chemotherapy. If chemotherapy is not planned, Day 1 should occur within three weeks of screening visit. . At the end of the 12 weeks, participants are invited to enrol in an extension phase where they will continue to receive blinded intervention medication for another 12 weeks. The optional extension (weeks 13-24) is entirely based on participant's preference.

All participants will receive standard physical activity and diet advice leaflets from the study nurse as part of a multi-modal intervention at baseline.

Participants will visit the study centre at baseline, weeks 4, 8 and 12 (plus 18 and 24 for those in the extension phase). During the visits, the study nurse will take the measures and assessments. Primary and secondary efficacy analysis will be conducted at 12 weeks. Additional exploratory efficacy and safety analyses will also be conducted at 24 weeks (for those who opt into the extension phase) in order to collect data over longer treatment duration.

3.2 Study Diagram

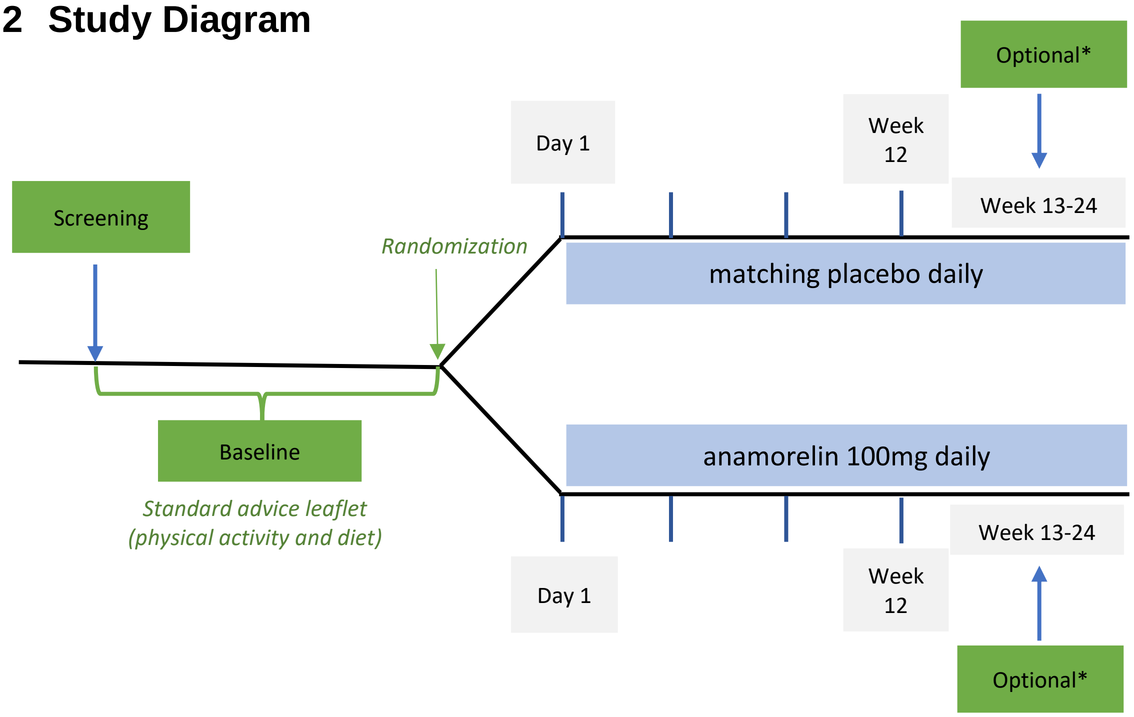

**The optional extension is entirely based on participants' preference for blinded continuation for weeks 13-24.*

3.3 Study setting

This is a multi-site study including sites across states in Australia. This includes, but is not limited to, New South Wales (NSW), Victoria (VIC) and South Australia (SA).

3.4 Study population

3.4.1 Target population

Adults (aged ≥18 years) newly diagnosed with small cell lung cancer with planned systemic therapy OR with first recurrence of disease following successful treatment with a documented disease-free interval of at least ≥ 6 months, AND with anorexia (i.e. ≤ 37 points on the 12-item Functional Assessment of Anorexia Cachexia Treatment (FAACT A/CS) scale).

3.4.2 Eligibility criteria

People who meet **all** of the inclusion criteria and **none** of the exclusion criteria are eligible to enter this study.

3.4.2.1 *Inclusion criteria*

1. ≥18 years of age.
2. Documented histologic or cytologic diagnosis of small cell lung cancer (limited – one lung and/or nearby lymph nodes; or extensive disease – extends beyond single lung, and extended to other lymph nodes or other parts of the body).
3. Newly diagnosed with small cell lung cancer with planned systemic therapy* OR first recurrence of disease following successful treatment with a documented disease-free interval of ≥ 6 months.
4. ≤ 37 points on the 12-item Functional Assessment of Anorexia Cachexia Treatment (FAACT A/CS) scale.
5. Australia-modified Karnofsky Performance status ≥50 at screening.
6. Adequate hepatic function [AST (SGOT) and ALT (SGPT) ≤ 5 x ULN].
7. Adequate renal function (calculated creatinine clearance > 20 mL/minute).
8. English-speaking (or have an interpreter available).
9. The participant must be willing and able to provide written informed consent, and comply with the protocol tests and procedures.
10. Female participants shall be**:
    a) of non-childbearing potential; OR
    b) of childbearing potential using reliable contraceptive measures AND having a negative urine pregnancy test at baseline prior to first dose of investigational product.

*Notes:

Planned therapy includes the period from having a defined cancer treatment schedule and study medication starting up to one day prior to day one of Cycle 2 of chemotherapy.

Under no circumstances should the study delay the routine treatment for SCLC.

**Notes:

I) Female participants of non-childbearing potential are defined as being post-menopausal for at least 1 year; or having documented surgical sterilization or hysterectomy at least 3 months before study participation.

II) Reliable contraceptive measures include implants, injectables, combined oral contraceptives, intrauterine devices, vasectomized partner or complete (long term) sexual abstinence.

3.4.2.2 *Exclusion criteria*

1. Women who are pregnant OR breastfeeding

2. Pathology and causes that may impede food intake, as determined by the Investigator. These causes may include but are not limited to:
    a) Grade 3 or 4 oral mucositis;
    b) Grade 3 or 4 GI disorders (nausea, vomiting, diarrhea, and constipation); OR
    c) mechanical obstructions making the person unable to eat.
3. Having undergone major surgery (central venous access placement and tumor biopsies are not considered major surgery) within 4 weeks prior to randomisation. Potential participants must be recovered from acute effects of surgery prior to screening. Participants should not have a current treatment plan to undergo major surgical procedures during the treatment period.
4. Currently taking androgenic compounds (including but not limited to testosterone, testosterone-like agents, oxandrolone, megestrol acetate, methylphenidate, corticosteroids), olanzapine, prokinetics (including metoclopramide), dronabinol or medical marijuana (medical cannabis) or any other prescription medication or off-label products intended to increase appetite or treat unintentional weight loss [e.g. melatonin, nabilone, delta-9-tetrahydrocannabinol (THC) and cannabidiol (CBD)] – With the exception when any of these medications are administered (short-term) as part of routine chemotherapy/ radiation therapy standard protocols.
5. On mirtazapine in the previous four weeks.
6. Pleural effusion requiring thoracentesis.
7. Pericardial effusion requiring drainage.
8. Oedema requiring regular diuretics.
9. Ascites requiring drainage.
10. Uncontrolled or significant cardiovascular disease, including but not limited to:
    a) history of myocardial infarction within the past 3 months;
    b) A-V block of second or third degree (but eligible if currently has a pacemaker – with the exception that BIA will not be performed if the person has a pacemaker, due to minor electrical current);
    c) unstable angina;
    d) congestive heart failure within the past 3 months, if defined as New York Heart Association (NYHA) class III-IV;
    e) any history of clinically significant ventricular arrhythmias (such as ventricular tachycardia, ventricular fibrillation, Wolff-Parkinson-White (WPW) syndrome, or *torsade de pointes*);
    f) uncontrolled hypertension (blood pressure >160 mmHg systolic and >100 mmHg diastolic);

g) heart rate < 50 beats per minute on pre-entry electrocardiogram and participant is symptomatic.

11. Taking regular medications that may prolong the PR or QRS interval durations, such as any of the antiarrhythmic medications Class I [fast sodium (Na) channel blockers (e.g. quinidine, disopyramide, procainamide, lidocaine, phenytoin, flecainide, propafenone)].
12. Unable to swallow oral tablets.
13. Severe gastrointestinal disease (including oesophagitis, gastritis, malabsorption).
14. History of a gastrectomy.
15. Recent history of radiotherapy of oesophagus area.
16. Diabetes mellitus with secondary organ dysfunction (coronary heart disease, previous stroke, renal insufficiency), or poorly controlled diabetes (patients with glycosylated haemoglobin – HbA1c >7% or hyperglycaemia – measured as a fasting blood glucose >7mmol/L or a random blood glucose >11mmol/L) despite receiving clinic-based diabetes care.
17. Diagnosis of anorexia caused by other reasons, as determined by the investigator such as:
    a) advanced AIDS;
    b) heart failure;
    c) uncontrolled thyroid disease.
18. Receiving strong CYP3A4 inhibitors (including clarithromycin, erythromycin, diltiazem, itraconazole, ketoconazole, ritonavir, verapamil, etc.) within 14 days of randomization.
19. Currently receiving tube feedings or parenteral nutrition (either total or partial).
20. Current use of excessive alcohol or illicit drugs.
21. Any condition, including the presence of laboratory abnormalities, which in the Investigator's opinion, places the potential participant at unacceptable risk if he/she were to participate in the study or confounds the ability to interpret study data.
22. Enrolment in a previous study with anamorelin HCl or previous exposure to anamorelin HCl.
23. Actively receiving a concurrent investigational agent or having received an investigational agent within 28 days of Day 1.
24. Cognitive impairment (Short Blessed Test (SBT) score ≥10).

Table 3-1 List of medications to check for exclusion criteria

Medication Class	Example of medications*
**Strong CYP3A4 Inhibitors**	clarithromycin
	erythromycin
	diltiazem
	itraconazole
	ketoconazole
	ritonavir
	verapamil
**Strong CYP3A4 Inducers**	Rifampicin
**Medications to prolong PR or QTS interval**	Antiarrhythmic medications Class I (fast sodium (Na) channel blockers (e.g. quinidine, disopyramide, procainamide, lidocaine, phenytoin, flecainide, propafenone)
**Androgenic compounds****	testosterone
	testosterone-like agents
	oxandrolone
	megestrol acetate
	methylphenidate
	corticosteroids
**Any other medication intended to increase appetite****	Delta-9-tetrahydrocannabinol (THC) and cannabidiol (CBD) or medical marijuana (medical cannabis)<br>dronabinol<br>Nabilone<br>Melatonin
**Other**	olanzapine<br>mirtazapine<br>prokinetics including metoclopramide

* These are only example of medications that should be checked during screening. Please note, the Investigator Brochure should be checked at all times.

** Exception when administered (short-term) as part of routine chemotherapy/ radiation therapy standard protocols.

3.4.3 Withdrawal criteria

Participants may withdraw their consent and discontinue the study treatment at any time. They may withdraw their consent for continued administration of the study medication with or without withdrawing consent from all protocol-specified assessments, follow-up procedures and for the study team to use already collected data. Every effort should be made to encourage participants to return for scheduled visits to undergo study procedures even if they discontinue the study drug. If participants withdraw study participation at any time,

they will be asked if they can participate in one final clinical visit and whether they would participate in a follow-up telephone call seven days later.

4.0 INTERVENTION

4.1 Study medication

- Group 1 / intervention arm – oral anamorelin HCl 100 mg once daily (administered as tablets in the fasted condition – ≥1 hour before first meal of the day).
- Group 2 / control arm – placebo once daily (administered as matching placebo tablets regarding appearance, smell and taste, in the fasted condition – ≥1 hour before first meal of the day).

At baseline, all participants will receive standardised and structured physical activity and diet advice leaflets from the study nurse as part of a multi-modal intervention. All participants will receive the same information delivered in the same way. All other therapies will continue as prescribed by the treating physician(s).

Details of the product information for anamorelin are provided in **Table 4-1**

Table 4-1 Product Information for anamorelin*

**Approved name**	Anamorelin
**Manufacturer of drug**	Helsinn Healthcare
**Supplier of drug**	Helsinn Healthcare
**Approved therapeutic indication, dosage/duration in Australia**	Not currently approved.<br>This study will be conducted through a CTN notification to the Australian Therapeutic Goods Administration (TGA)
**Believed mode of action**	Selective ghrelin receptor agonist
**Dosage regimen**	Orally once daily
**Mode of excretion**	Most of the drug is excreted in the faeces (92%).[17]
**Known adverse events**	Two phase III clinical trials in patients with advanced lung cancer have demonstrated that anamorelin was well tolerated. The most common drug-related treatment emergent adverse events were diabetes and hyperglycaemia. Grade 1 or 2 nausea was experienced by more patients assigned to anamorelin compared to placebo.[13]
**Known contraindications or warnings**	*Special warnings and precautions for use:* [18]<br>Blood Glucose: Monitoring of blood glucose during treatment with anamorelin, particularly in diabetic patients or in patients with impaired glucose tolerance, is recommended as blood glucose level may increase following anamorelin administration. In patients with

	pre-existing diabetes mellitus an assessment of the individual benefit-risk balance should be performed.<br>Hepatic impairment: Anamorelin should be used with caution in patients with mild to moderate hepatic impairment as its use in patients with hepatic impairment may be associated with increased exposure to anamorelin.<br>*Contra indications*:<br>Interactions with CYP3A4: Co-administration of anamorelin with medicinal products that inhibit (e.g ketoconazole, clarithromycin, erythromycin, diltiazem, itraconazole, ritonavir, verapamil) or induce (e.g. rifampicin) CYP3A4 activity may affect anamorelin plasma concentrations. Concomitant administration of anamorelin with strong CYP3A4 inhibitors should be avoided and the concomitant use of anamorelin with moderate CYP3A4 inhibitors should be done with caution. Concomitant administration of anamorelin with strong CYP3A4 inducers should be done with caution.<br>Food effect: Food decreases anamorelin bioavailability and consequently anamorelin should be taken on an empty stomach at least one hour before a meal.<br>Cardiac effects: Anamorelin induced prolongation of PR and QRS intervals at supra-therapeutic doses. Therefore, anamorelin should be used with caution in the case of co-administration of medicinal products that may increase its exposure such as antiarrhythmic medications Class I (fast sodium (NA) channel blockers (e.g. quinidine, disopyramide, procainamide, lidocaine, phenytoin, flecainide, propafenone).

**Further information is in the investigator brochure.*

4.2 Dosing schedule

The first day of dosing will be Day 1. The intervention will be delivered as one blinded tablet: one anamorelin HCl 100 mg tablet or one matching placebo tablet. Tablets of study drug are to be taken orally, once daily by the participant in mornings while fasting at least 1 hour before the first meal of the day. Water is permitted prior to and with study drug. Participants will take the first dose of intervention on Day 1, which is the day after receipt of the medication. If a fasting state is not possible on Day 1, participants will start study drug on the next possible day and daily thereafter until Week 12 or up to week 24, if a participant opts in for blinded continuation in the extension phase. If participants have missed any doses, irrespective of the number of doses missed, they will be instructed to not catch up the missed doses if the missed dose was more than 8 hours earlier, but to continue taking on the next day of treatment. Participants will be supplied with study drug within business days after randomisation (i.e. up to one day prior to starting Cycle 2 of chemotherapy) and at Week 18.

4.3 Method of assigning participants to treatment groups

At each site, people referred to the study will be sequentially allocated a unique identification (ID) number. This ID number will be used for all subsequent study documentation for that participant.

Treatment for each participant will be allocated in a 1:1 ratio. Block randomisation will occur in each stratum at each site in randomly assigned blocks of 4 or 8 and will ensure even allocation to each code. Central randomisation will be stratified by limited versus extensive disease and by baseline score of the 5-item Anorexia Symptom Scale Domain from the 12-item FACCT A/CS (≤10 vs >10). The 5-item from the FACCT A/CS are as follows: *F1. I have a good appetite; F6. My interest in food drops as soon as I try to eat; F7. I have difficulty eating rich or "heavy" foods; F10. When I eat, I seem to get full quickly; F4. Most food tastes unpleasant to me*.[55]

On notification of a participant, the delegated pharmacist at a third party pharmacy will email the ITCC Central Randomisation Service (Randomisation@uts.edu.au) providing the required registration details and receive the treatment allocation via reply email. This allocation will be printed or saved electronically into the pharmacy records. The participant ID, allocation code, dates of request, preparation, and dispensing will be recorded in a log maintained by the pharmacist and supplied to the central registry on each randomisation.

Participant randomisation will be registered with the IMPACCT Trials Coordination Centre (ITCC). In summary, the procedure outlines that, on randomisation of a participant, the third party pharmacy is to email a notification to the ITCC. This notice will be monitored alongside the participant eligibility as entered onto the electronic Case Report Form (eCRF) from the Eligibility data collection worksheet.

4.4 Blinding

Treatment allocation will not be disclosed to participants, study staff, treating clinicians or investigators. The code will only be broken in cases of extreme emergency (refer to 4.5).

The drug manufacturer will ensure study drug and placebo appear identical in regards to appearance, smell and taste. All medication will then be secondary packaged and dispensed in blinded packaging on a per participant basis by a contracted third party pharmacy, according to the treatment allocation derived from the randomisation schedule (refer to 4.6).

Each medication will be numbered according to the pre-determined allocation code and labelled as: *'No. 044/21 Anamorelin (100 mg), placebo. Take one capsule every morning one hour prior to the first meal until finished.'*

4.5 Unblinding procedures

The code will only be broken in cases of extreme emergency. Such situations only include where knowledge of the allocation will have consequences for clinical decision making in consultation with the Lead Investigator who will have access to the sealed unblinding schedule. The Lead Investigator, therefore, must be contacted in the first place. Clinical staff will be able to discuss the clinical situation with the Lead Investigator to determine the urgency and need for unblinding and will be informed by the Lead Investigator of the treatment allocation based on these discussions.

If the Lead Study Investigator is unable to be contacted, the ITCC National Manager should be contacted.

The ITCC will monitor real time unblinding frequency by collecting eCRF withdrawal data and unblinding reporting from the Lead Study Investigator.

4.6 Drug supply

The drug manufacturer will provide the bulk tablets (active and matching placebo). A licensed local pharmaceutical development and manufacturing organisation contracted by the Sponsor will import and store the bulk supply. This will be made available on a Just-in-Time basis to their sub-contracted central pharmacy (referred to as third party pharmacy) for the dispensing activity. The third party pharmacy will secondary package the bulk tablets into study medication kits (90 tablets/kit for the first 12 weeks of treatment period and 80 tablets/kit for the extension phase).The third party pharmacy, will dispense the study medication on a per participant basis upon receipt of a valid prescription. A log will be maintained of the kit dispensed to each participant.

All medications must be dispensed in accordance with the Just in Time delivery system used within the third party pharmacy. The third party pharmacy will dispense the study medication directly to the participant at their home on a per participant basis. The third party pharmacy will retain the randomisation and investigational product dispensing and accountability responsibilities.

4.7 Drug accountability and destruction

All active medicine and placebo must be stored in a locked medicine cabinet at or below 25°C within the pharmacy. The pharmacy will maintain accountability records appropriate to State regulations, in addition to the study allocation records.

Participants will be instructed to bring their diary to each study visit during the treatment period for a compliance/accountability check. Compliance will be assessed at visits at weeks 4, 8 and 12 (plus 18 and 24 for those in the extension phase) using participant record of self-administration (i.e. participant diary) OR counting of the remaining tablets at treatment cessation, if the information has not been recorded in the participant diary. A participant will be considered to be compliant with investigational medication if she/he takes all study drugs provided. If participants have missed any doses, irrespective of the number of doses missed, they will be instructed to not catch up the missed doses if the missed dose was more than 8 hours earlier, but to continue taking on the next day of treatment.

After the last dose is taken and study medication is ceased, participants will return all unused study medication to the contracted licensed pharmaceutical development and manufacturing organisation using the return-addressed satchel provided to the patient with the dispensed drug. Upon receipt of the unused study medication, final accountability will be completed and documented in the study pharmacy records.

All unused study medication will be destroyed in a manner consistent with the applicable regulations governing destruction in each state or country. The contracted licensed pharmaceutical development and manufacturing organisation Standard Operating Procedures and state regulations are to be referred to and adhered to at all times.

4.8 Cessation of study drug/intervention

Treatment may be ceased for one of the reasons listed below. A description of the reason behind ceasing the drug will be recorded in the medical record or clinical file as per the site's SOP.

- Reached primary endpoint (12/ 24 weeks).
- Participant request.
- Insertion of tube feeding or parenteral nutrition.

- Unacceptable side effects from study medications (defined by National Cancer Institute Common Criteria for Adverse Events; NCI-CTCAE version 5.0) of grade 3 not resolving with appropriate treatment or any grade 4 or 5 toxicities.
- An AE occurs that, in the opinion of the Investigator, makes it unsafe for the participant to continue in the study.
- Participants who in the opinion of the Investigator are not well enough to continue the study (specific reasons for cessation need to be documented in the data collection worksheet and electronic Case Report Form – eCRF).
- It is inappropriate to continue the study medicine for whatever reason.
- The participant or proxy withdraws their consent for continued administration of the study medication, with or without withdrawing consent from study and all protocol-specified assessments and follow-up procedures.
- The participant dies.
- The participant is lost to follow-ups.
- The Investigator, for any reason, terminates the entire study, or terminates the study for that participant; or the attending physician requests that the participant be withdrawn for any medical reason.
- The Sponsor or the Regulatory Authorities or the Ethics Committee(s), for any reason, terminates the entire study, or terminates the study for this trial site or participant.

If a participant prematurely discontinues treatment with anamorelin/placebo at any time prior to completing the 12-week visit, the Investigator must try his/her best to contact the person for scheduled visits. Every effort will be made to collect data from the protocol-specified assessments. Participants will be asked to continue to participate in the study and complete planned visits up to Week 12 even if they have permanently discontinued treatment with anamorelin/placebo ("retrieved dropout" approach). If participants discontinue study participation at any time, they will be asked if they can participate in one final clinical visit and whether they would participate in a follow-up telephone call seven days later.

4.9 Treatment failure

The circumstances listed below will be considered a treatment failure. A description of the treatment failure will be recorded in the medical record or clinical file as per the site's SOP.

- Adverse events related to the study medicine unacceptable to participant, caregiver or clinician in charge;
- Treatment deemed ineffective by treating clinician, who wishes to use alternative therapy;
- Other situations that may require cessation of study medicine:
    - Extended non-response.

4.10 Stopping rules

The study will be stopped if treatment failure is considered (refer to 4.9) and new literature indicates findings that can be applied to this question in terms of benefit or side effects, or if reporting of adverse events (AEs) indicate that review of the study protocol is required.

5.0 OUTCOMES AND MEASURES

5.1 Primary Outcome

5.1.1 Feasibility

Progression criteria will be assessed using an indicative traffic light system (green, orange red) whereby green (go) suggests that the criteria have been met and the trial should proceed, orange (amend) indicates that some changes should be made to the larger trial and red (stop) indicates that a larger trial should not proceed. Feasibility will be assessed by evaluating:

1. **Recruitment rate,** defined as an average of one participant per site (six sites) every six weeks – plan to recruit 50 participants.
2. **Proportion of participants with complete primary endpoint data** – >70% (green), 60-70% (orange), <60% (red).
3. **Participant adherence to investigational medication** – >70% (green), 60-70% (orange), <60% (red).
4. **Participant adherence to physical activity advice,** as measured by the Short Questionnaire to Assess Health Enhancing Physical Activity (SQUASH), completed at every visit (whereby increase in physical activity will be reviewed over the study period) – >40% (green), 30-40% (orange), <30% (red).
5. **Participant adherence to dietary advice,** as measured by the food diary kept for three days prior to visits (whereby increase in macronutrients intake will be reviewed over the study period) – >40% (green), 30-40% (orange), <30% (red).

5.1.2 Desirability

The study will be considered desirable if there is sufficient signal in the efficacy evaluation to suggest that an adequately powered study would deliver a clinically meaningful difference between groups (where the primary measure has a minimum clinically important difference pre-defined), feasibility end-points are met and participants find the study acceptable.

5.1.3 Efficacy (for clinical assessments)

1. Change in the 5-item Anorexia Symptom Scale Domain from the 12-item Functional Assessment of Anorexia Cachexia Treatment (FAACT, anorexia/cachexia – A/CS) scale by arm from baseline to week 12.
2. Rates of *on time, on dose* completion of any administered chemotherapy and/or radiotherapy for SCLC during the duration of the study.

5.2 Secondary endpoints

5.2.1 Secondary efficacy (clinical assessments proposed for any subsequent phase III study)

1. Baseline demographic and clinical predictors of responders.
2. Change in body weight and BMI at every visit and assessment of historical stable weight at baseline.
3. Change in lean body mass measured at baseline and week 12 (or 24) by dual x-ray absorptiometry (DEXA) and Biolectrical Impedance Analysis (BIA).
4. Change in muscle mass at thoracic (T4) level, measured by electronically stored data from computed tomography (CT) scans whenever they are done clinically.
5. Functional status using clinician-rated Australia-modified Karnofsky Performance Scale (AKPS) measured as area under the curve from data collected at every scheduled visit.
6. Timed *up and go test* (TUG) measured at every scheduled visit.
7. Functional performance as measured by a pedometer app (stepapp) or a wearable digital pedometer, for participants who do not have mobiles, worn for two days (preferably one weekday and one weekend day) prior to visits.
8. Nutritional intake measured by self-reported 3-day food diary, including 2 weekdays and one weekend day prior to visits. If participants do not complete the 3-day food diary prior to visits, a 24-h diet recall will be collected by the study nurse at the time of visit.
9. Biochemical changes including C Reactive Protein (CRP), IL-6, albumin and pre-albumin from baseline.
10. Survival (overall and cancer-specific) measured with data of death from the time of enrolment.
11. Changes overtime in the participant rated Global Impression of Change (GIC) and in the clinician rated Clinical Global Impression of Severity and Improvement (CGI-S, CGI-i).
12. Changes overtime in quality of life measured by EQ-5D-5L (EuroQoL five-dimensional instrument) and ICECAP-A (ICEpop CAPability measure for Adults).

13. Changes overtime in participant-reported fatigue as measured by the Functional Assessment of Chronic Illness Therapy-Fatigue (FACIT-F) domain.
14. Rates of unplanned hospitalisations (defined as an emergency admission; or unplanned emergency contact with GP, nurses, specialists, home help visit, etc.; or unplanned readmission within 28 days following discharge to the same facility – participant may arrive at hospital in own transport or in an ambulance) and length of stay; with primary reasons kept in a diary by participants.
15. Episodes of febrile neutropaenia will be collected from participant's medical record.
16. Adverse events from chemotherapy (nausea, vomiting, anorexia, fatigue) will also be collected from participant's medical record.
17. Harms assessed in each visit.

5.2.2 Safety Assessments

Safety assessments are made at all participant contacts, before efficacy assessments. The following safety assessments will be obtained during the study:

1. physical examination (PE);
2. vital signs;
3. 12-lead electrocardiogram (ECG);
4. laboratory tests (hematology and blood chemistry);
5. documentation of adverse events (harms);
6. overall survival on all participants; and
7. tumour assessments using CT scans whenever they are done clinically using RECIST (response evaluation criteria in solid tumours) criteria[19].

5.3 Tertiary endpoints

5.3.1 Cost effectiveness and caregivers' assessments

1. Changes in caregiver's quality of life measured by EQ-5D-5L and unmet needs measured by CES (Carer Experience Scale).

2. Capture participants and caregivers indirect and informal care costs and resource use (e.g. time off work, time spend in-home nursing or accompanying to hospital, any out-of-pocket expenses) kept in a diary by participants.

6.0 STUDY ASSESSMENTS

The study assessments are tabulated in Table 6-1. The study period will include 28 days after medication cessation (4 weekly follow-up period) or until death (whichever timeframe is shorter).

6.1 Laboratory measures

Venous blood samples (approximately 9mL/assessment) will be drawn for screening and follow up assessments. In some instances, blood samples taken as part of diagnosis and all pre-chemo/radiation therapy blood works can be used for screening, baseline and follow-up assessments if the clinical picture has not otherwise changed within that time. The results will be held in the participant study file as source data.

Samples for safety laboratory assessments will be taken at pre-set time points as specified in the Assessment Schedule and participant timeline. All tests require no fasting period for participants. All samples will be sent to the local hospital laboratory for analysis.

Haematology

The haematology assessments will include full blood count (FBC) with differential count and will be performed at: screening visit (Visit 1), Week 4 (Visit 3) and Week 12 (Visit 5) as well as during optional extension phase of this study at Week 24 (Visit 7).

Blood Chemistry

Blood Chemistry will include sodium, potassium, chloride, calcium, total protein, albumin and pre-albumin, aspartate aminotransferase [AST (SGOT)]*, alanine transaminase [ALT (SGPT)]*, alkaline phosphatase (ALP), total bilirubin, creatinine [creatinine clearance* will be calculated using the modification of diet in renal disease (MDRD) formula (the current Australian national standard)[21]], haemoglobin A1c (HbA1c) and random glucose**. These will be performed at the screening visit (Visit 1), Week 4 (Visit 3) and Week 12 (Visit 5) as well as during the optional extension phase of this study at Week 24 (Visit 7).

In addition, blood samples for IL-6 will be collected at baseline (Visit 2), Week 4 (Visit 3) and at end of treatment (i.e. either Week 12 (Visit 5), Week 24 (Visit 7) or at Withdrawal).

C-reactive protein (CRP) will be collected at baseline (Visit 2), Week 4 (Visit 3), Week 12 (Visit 5) as well as during the optional extension phase of this study at Week 24 (Visit 7). C-reactive protein will be used to assess the level of infection or inflammation in the body.

*Notes: Only patients with a calculated creatinine clearance of >20ml/min will be eligible to participate in the study. Similarly, liver function, as measured by AST (SGOP) and ALT (SGPT) will be assessed prior to entry into the study, with an expected result of ≤ 5 x ULN for eligibility.

**Notes: All participants with diabetes enrolled in this study will be required to monitor their blood glucose levels in consultation with their treating physician. People who are insulin dependent will be encouraged to commence daily monitoring (if not already doing so) in consultation with their treating physician. During their participation in the study, blood glucose levels and HbA1c concentrations will be assessed as part of blood chemistry at specified study visits [including, screening visit (Visit 1), Week 4 (Visit 3), Week 12 (Visit 5) and at Week 24 (Visit 7, if applicable)]. Changes in blood glucose levels will be reviewed as adverse events.

Pregnancy Screening

Urine pregnancy testing for women of childbearing potential will be performed at screening visit (Visit 1), baseline (Visit 2) and Week 12 (Visit 5).

A negative urine pregnancy test will be needed at baseline prior to first dose of investigational product.

6.2 Clinical and physical measurements

Height in centimetres (cm) will be collected at screening (Visit 1). The patient’s height will be recorded to the nearest 0.5 cm while the patient is barefoot and standing.

Body weight in kilograms (Kg) will be collected at every visit: screening visit (Visit 1) and baseline (Visit 2), Weeks 4, 8 and 12 (Visits 3, 4 and 5) as well as during optional extension phase at Weeks 18 and 24 (Visits 6 and 7). Clinical studies have shown that anamorelin administration resulted in an increase in body weight in patients with NSCLC and anorexia/cachexia[22] hence, body weight will be recorded in this study in order to understand if a similar trend is observed for patients with SCLC and anorexia. In addition, historical stable weight will be gathered by self-reported weight (in Kg) in the last 1, 6 and 12 months collected at screening visit (Visit 1). A scale will be supplied to each participating site for consistency of data collected and to avoid miscalibrations.

Body mass index (BMI) will be calculated from the patient's weight divided by the patient's height squared.

Body composition will be obtained using dual x-ray absorptiometry (DEXA) and the lean body mass and fat mass will be recorded. In addition, bioelectrical impedance analysis (BIA), a simple, non-invasive, low-cost method will also be used to estimate body composition. These will be done at baseline (Visit 2) and at end of treatment, i.e. at Week 12 (Visit 5) (for those electing not to enter the extension phase) or Week 24 (Visit 7) for those participating in the extension phase, or at withdrawal, whichever occurs first. All measurements will be performed at a standardised time of day (morning or afternoon). Scales for BIA will be provided to each participating site for consistency. Furthermore, muscle segmentation in axial computed tomography (CT) images at the thoracic (T4) level will be obtained from all electronically stored data from CT scans whenever they are done clinically as part of usual care. Clinical studies have shown that anamorelin administration resulted in an increase in lean body mass in patients with NSCLC and anorexia/cachexia[22]. Hence lean body mass will be assessed in this study in order to understand if a similar trend is observed for patients with SCLC and anorexia.

A ***complete physical examination*** (PE) of the patient will be completed at the initial screening (Visit 1) to obtain a medical assessment of the patient's general condition and at follow up visits (Visits 3, 4, 5 and 6 and 7, if applicable). This evaluation will include an examination of general appearance, head, eyes, ears, nose, throat, skin, neck, lungs, cardiovascular, breast, lymph nodes, abdomen, musculoskeletal and neurological. The medical examination will also include a complete cardiac history to assess for previous or recent cardiac events, and will make use of physical examination, close review of the medical record, and assessment of the New York Heart Association Classification, if appropriate.

In addition, a ***dedicated physical exam*** aiming to monitor signs and symptoms of **oedema** such as pedal oedema, pericardial effusion, pleural effusions, and ascites will be conducted at screening (Visit 1) and follow up visits (Visits 3, 4, 5 and 6 and 7, if applicable). Limb oedema (tibia, feet including ankles, and hands) should be evaluated for pitting (and graded 0-4+), and whenever possible, for tenderness to palpation (by applying pressure), skin temperature, colour, and texture changes. This will be done since excess in body water may contribute to a change in body weight and confound the primary efficacy analysis. PE of limbs should focus on the dorsum of the foot, all foot including ankle, the medial malleolus, the bony portion of the tibia. PE of ascites, pleural and pericardial effusions should focus on visual and palpation assessment of the abdominal area and evaluation of thoracic/pericardial oedema will be complemented by auscultation.

Information about the physical examinations will be recorded in the source documentation at the site and any abnormalities at screening will be recorded in the eCRF. After signing the informed consent, development or worsening of existing pathological findings noted since previous examination during the study will be reported on the AE eCRF form.

Vital signs including body temperature (◦C), blood pressure (mmHG, systolic and diastolic), respiratory rate (breaths per minute) and heart rate (beats per minute) will be measured as part of the safety assessments at every visit: screening (Visit 1) and at Weeks 4, 8 and 12 (Visits 3, 4 and 5) as well as during optional extension phase at Weeks 18 and 24 (Visits 6 and 7). Pulse rate, systolic and diastolic blood pressure will be measured after the patient has been resting in the semi-supine position for at least 5 minutes.

A ***complete medical history*** will be recorded at the screening visit (Visit 1) and will include evaluations for past or present conditions. **Concomitant medications** will also be recorded at every visit: screening (Visit 1), baseline (Visit 2) and at Weeks 4, 8 and 12 (Visits 3, 4 and 5) as well as during optional extension phase at Weeks 18 and 24 (Visits 6 and 7).

12-lead ECGs will be conducted at screening (Visit1), Weeks 4, 8 and 12 (Visits 3, 4 and 5). Any new or worsening ECG abnormalities assessed to be clinically significant by the study investigator will be reported as adverse events. When possible, ECG will be recorded at 1 hour (±5 minutes) post dose to align with occurrence of Cmax. At screening visit ECG will be taken pre-dose (as no dose will be administered at this time).

Short Blessed Test will be administered at screening to assess cognitive capacity and will assist the PI to assess eligibility by determining the persons capacity to understand the study and to complete the assessments. A score of 10 or more is required.

Adverse event assessment the research team member who sees the participants and their caregivers will also ask about any other unexpected adverse outcomes. The study investigators will oversee the research team members. All serious adverse events will be reported in accordance with the requirements of the approving central Research and Ethics Committee as defined in the committee's safety reporting standard operating procedures (see section 9.1 Adverse Events). Serious adverse events will be followed until documentation of resolution or the successful initiation of relevant management strategies. Other adverse events will be described in the Annual Report to the Committee.

Clinical Global Impression (CGI)

The CGI is a two-item clinician-rated questionnaire designed to assess impression of disease severity (CGI-S) and global improvement or change (CGI-i) with a seven-point scale (1-7) - 1 = "Normal or not ill" 2 = "Borderline ill," 3 = "Mildly ill," 4 = "Moderately ill" 5 = "Markedly ill," 6 = "Severely ill," or 7 = "Among the most extremely affected subjects"; and 1 = "Very much improved" 2 = "Much improved," 3 = "Minimally improved," 4 = "No change" 5 = "Minimally worse," 6 = "Much worse" or 7 = "Very much worse", respectively."[29] CGI-S will be filled out at baseline (Visit 2) and Weeks 4, 8 and 12 (Visits 3, 4 and 5) and at Week 24 during the optional extension phase (Visit 7). CGI-i will be filled out at Weeks 4, 8, 12 (Visits 3, 4 and 5) and at Week 24 (Visit 7) if applicable.

6.3 Comorbidity assessment

Comorbid conditions will be recorded using the Charlson Comorbidity Index which incorporates in a single score the severity and number of comorbid conditions [23, 24]. The index is an independent predictor of long-term survival (predicting the 10-year survival in patients with multiple comorbidities), and although long-term mortality may not be the most important factor in this study, it allows a uniform measure of comorbidity in a population where this may be frequently encountered. The Charlson Comorbidity Index will be used without weighting.

6.4 Demographics

1. Age and date of birth
2. Biological gender
3. Postcode
4. Language spoken at birth and at home
5. Ethnicity, including Aboriginal or Torres Strait Islander status
6. Smoking history/ smoking status
7. Country of birth

6.5 Main clinical diagnosis

Relevant information on tumour type, staging (limited vs. extensive), date of diagnosis, sites of metastasis and cancer treatment received and planned (including when available, relevant eVIQ protocol number) will be collected at the screening (Visit 1). The extent of disease will be recorded as limited or extensive for the purposes of stratification.

Small cell lung cancer treatment regimen and any dose or interval adjustments will also be collected at relevant follow up visits during cancer treatment, including baseline and Weeks 4, 8 and 12 (Visits 2, 3, 4 and 5), and 18 and 24 (Visits 6 and 7, if extension phase). In addition, response to therapy for target lesions of SCLC will be collected at Weeks 12 (Visit 5) and 24 (Visit 7, if extension phase).

6.6 Functional status

Australia - modified Karnofsky Performance Status

The Australia-modified Karnofsky Performance Status (AKPS) is a validated variant of the Karnofsky Performance Status scale[25]. The Australian version has criteria that can be applied in either the inpatient or outpatient setting and reflects the greater emphasis on community-based care. This objective measure has high inter-rater reliability and is sensitive to changes in function over time. A score of 0 to 100 (in increments of 10) is assigned to participants based on their ability to undertake a range of daily tasks. The score gives an indication of the participant condition (in terms of physical ability) and can assist in prognostication. The tool will be used in this study to assist investigators to determine participant condition and possible prognosis, together with any measurable improvements in functional status as a result of the intervention. The AKPS will be collected at every visit: screening (Visit 1), baseline (Visit 2), and at Weeks 4, 8 and 12 (Visits 2, 3, 4 and 5) as well as during optional extension phase at Weeks 18 and 24 (Visits 6 and 7).

Step count

A downloadable pedometer app (*stepapp* – available for free) will be used to objectively track physical activity (step count) of patients during the study. The mobile app should be used at all times over 2 days (preferably one weekday and one weekend day) immediately prior to visits during baseline, Weeks 4, 8 and 12, and at Weeks 18 and 24 for the optional extension. Participants who do not have mobiles, will be given a wearable digital pedometer. Participants will be asked to enter daily step count on a diary. Differences in absolute and percentage change for these data will be analysed.

Timed up and go test

The Timed Up and Go (TUG) test is a functional mobility test that requires the patient to stand up, walk 3 metres, turn, walk back and sit down.[26] The presence of slowness, hesitancy, abnormal trunk or arm movements, staggering or stumbling is used to grade the patient from 1 (normal) to 5 (severely abnormal). A score of 3 (mildly abnormal) or more suggests a risk of falling. A slow time suggests an increased risk of falls. The test will be conducted at: baseline (Visit 2), and Weeks 4, 8 and 12 (Visits 3, 4 and 5), and at Weeks 18 and 24 for those participating in the optional extension phase (Visits 6 and 7).

6.7 Patient reported symptom measure

Functional Assessment of Anorexia/Cachexia Therapy (FAACT)

The functional assessment of anorexia/cachexia therapy (FAACT) questionnaire is an instrument for diagnosing anorexia[27]. There are 12 items on the FAACT that are related to the patient's appetite in the last seven days. Each item is scored on a five-point Likert scale (zero equates to not at all and four equates to very much). From this questionnaire the 5-item section referring to anorexia symptoms will be used to assess primary efficacy endpoint. The assessment will be filled out by patients at every visit: screening (Visit 1), baseline (Visit 2) and at Weeks 4, 8 and 12 (Visits 3, 4 and 5) as well as during optional extension phase at Weeks 18 and 24 (Visits 6 and 7).

Global Impression of Change

This participant rated seven-point scale (1-7) provides information about the participant perception of their change in clinical status, specifically their improvement since the commencement of the study. 1 = "Very much improved" 2 = "Much improved," 3 = "Minimally improved," 4 = "No change" 5 = "Minimally worse," 6 = "Much worse," or 7 = "Very much worse." The results of this scale over the study period will assist to determine the clinical significance of any improvement seen[28]. The assessment will be filled out by patients at Weeks 4, 8 and 12 (Visits 3, 4 and 5) and at Week 24 during the optional extension phase (Visit 7).

EuroQol 5-Level 5-Dimensions – Quality of Life

Quality of life of participants will be measured using the EQ-5D-5L[30]. There is currently no single agreed measure of health-related quality of life in palliative care populations. EQ-5D-5L assesses mobility, self-care, usual activities, pain / discomfort, anxiety / depression according to three levels of severity (1 = no problems; 2 = some

or moderate problems; 3 = extreme problems), plus a VAS of current health-related quality of life, scored 0 – 100[31-33]. This will be administered to patients at baseline (Visit 2), Weeks 8 and 12 (Visits 4 and 5) as well as during optional extension phase at Week 24 (Visit 7).

ICECAP-A – ICEpop CAPability measure for Adults

ICECAP-A is a measure of capability for the general adult (18+) population for use in economic evaluation. It focuses on wellbeing defined in a broader sense, rather than health and comprises five attributes: attachment (ability to have love, friendship and support), stability (ability to feel settled and secure), achievement (ability to achieve and progress in life), enjoyment (ability to experience enjoyment and pleasure) and autonomy (ability to be independent).[34, 35] The ICECAP-A will be administered to the participants after the ED-5D-5L questionnaire at bassline (Visit 2) and Weeks 8 and 12 (Visits 4 and 5), as well as during optional extension phase at Week 24 (Visit 7). It will probe valuable additional, symptom-specific information to the EQ-5D-5L. Its use in the study may also give insight into which QoL questionnaire participants find more acceptable.

Short Questionnaire to Assess Health Enhancing Physical Activity – SQUASH

Physical activity undertaken and participant adherence to physical activity advice will be recorded by patients using the Short Questionnaire to Assess Health Enhancing Physical Activity – SQUASH. This tool was developed to give an indication of the habitual activity level.[36] The SQUASH contains questions on multiple domains referring to an average week in the past month: commuting activities (walking, cycling), household activities, leisure time (sports, walking, gardening, cycling) and activities at work or school. The assessment will be filled out by patients at baseline (Visit 2), Weeks 4, 8, and 12 (Visits 3, 4 and 5) as well as during the optional extension phase at Weeks 18 and 24 (Visits 6 and 7). Increase in physical activity will be reviewed over the study period. A traffic light system for progression criteria assessment will be used whereby >40% indicates that the criteria have been met (green), 30-40% indicate that some changes should be made to the larger trial (orange) and <30% indicates that a further trial should not proceed (red).

Functional Assessment of Chronic Illness Therapy-Fatigue (FACIT-F)

Fatigue is a disruptive symptom that inhibits normal functional performance. The functional assessment of chronic illness therapy-fatigue (FACIT-F) scale is a tool that measures the individual's level of fatigue during their usual daily activities over the past week.[37]The FACIT-F scale is a 13-item scale and the level of fatigue for each item is measured on a four point Likert scale (four equates to not at all fatigued and zero equates to very much

fatigued), the range of possible scores is 0-52. The assessment will be filled out by participants at baseline (Visit 2) and Weeks 8, and 12 (Visits 4 and 5) as well as during the optional extension phase at Week 24 (Visit 7).

Participant diary

Participants will be asked to record daily step counts as described above. Adherence to dietary advice is a feasibility measure in this trial and will be assessed by asking participants to keep a food diary of everything they eat and drink for 3 complete days preferably prior to study visits, including, when possible, 2 weekdays and 1 weekend day (e.g. Thursday, Friday and Saturday). Participants will be encouraged to bring their complete food tracking diary during each visit. If participants do not complete the 3-day food diary prior to visits, a 24-h diet recall will be collected by the study nurse at the time of visit. Any increase in macronutrient intake will be reviewed over the study period. A traffic light system for progression criteria assessment to a larger phase 3 study will be used whereby >40% indicates that the criteria have been met (green), 30-40% indicate that some changes should be made to the larger trial (orange) and <30% indicates that a further trial should not proceed (red). The contents of the food diary will be entered into a dietitian directed database in order to analyse if the food intake recorded within the diaries complies with the dietary advice provided to each participant.

Unplanned healthcare costs.

Participants (and caregivers) will also be reminded to record in their diaries resource use to estimate cost of indirect and informal care. This will help assess the proposed measures for a health economic analysis (cost consequence) in any subsequent phase III study. Questions including time off work for patients, caregiver time spend in-home nursing or accompanying to hospital, out-of-pocket expenses, etc. Furthermore, participants will also be asked to record any unplanned healthcare contact/ hospitalisations defined as an emergency admission; or unplanned emergency contact with GP, nurses, specialists, home help visit, etc.; or unplanned readmission within 28 days following discharge to the same facility. These include inpatient/outpatient attendance and community care and participant may arrive at hospital in own transport or in an ambulance. Length of stay with primary reasons will also be asked to be entered in a diary by participants from baseline to Week 12 (or 24).

Participant acceptability of the study and proxy response measurement

Participant's acceptability of the study is a primary endpoint under desirability. This will be assessed at the time of participant's last visit (Week 12 or Week 24 if the patient opts in to the study extension phase). A set of 3 questions will be asked to evaluate if participants find the study acceptable. In addition, information on whether

participant's required assistance to complete study tools will be evaluated by one screening question and two follow up questions if they answer 'No' to the screening question.

6.8 Caregiver reported measure

Carer Experience Scale

The Carer Experience Scale (CES) is a profile measure of the caring experience for use in economic evaluation.[38, 39] It focuses on 'care-related quality of life' rather than health-related quality of life, comprising attributes that are pertinent to unpaid carers. The tool comprises six attributes: activities, support from family and friends, assistance from organisations, fulfillment, control, and getting-on with the care recipient. This will be administered to caregivers at the start and cessation of treatment, i.e. at baseline (Visit 2) and Week 12 or 24 (Visits 5 or 7, if patient opts into extension phase).

EuroQol 5-Level 5-Dimensions – Quality of Life Caregiver

Quality of life of caregivers will be measured using the EQ-5D-5L[30]. EQ-5D-5L assesses mobility, self-care, usual activities, pain / discomfort, anxiety / depression according to three levels of severity (1 = no problems; 2 = some or moderate problems; 3 = extreme problems), plus a VAS of current health-related quality of life, scored 0 – 100[31-33]. This will be administered to caregivers at the start and cessation of treatment of the person they provide care for, i.e. at baseline (Visit 2) and Week 12 or 24 (Visits 5 or 7, if patient opts into extension phase).

Caregiver diary

Caregivers will be asked to record basic demographic and caregiver details once only at baseline and a repeated series of questions on completion of the study. This will enable changes in the caregiving needs to be assessed.

Unplanned healthcare costs.

Caregivers will also be asked to include resource use to estimate cost of indirect and informal care in their diary. This will help assess the proposed measures for a health economic analysis (cost consequence) in any subsequent phase III study. Questions including time off work, time spend in-home nursing or accompanying to hospital, out-of-pocket expenses, etc.

6.9 Participant retention and follow-up

Evidence of the study's responsiveness to participant needs and flexibility is critical for participant retention.

Study staff are trained to expect that at times, it will be necessary to reschedule evaluations and appointments.

Ensuring that the study involves minimal burden to participants is crucial in this.

The study assessments are tabulated below.

Table 6-1 Schedule of Study Assessments for patients (Screening to Week 12 and optional extension – Weeks 13-24)

Assessment	Screening	Baseline	Visit Day Treatment Period			Extension		Exit/ Early Termination (Withdrawal)[a]	Follow-up[b]
**Week**			**4**	**8**	**12**	**18**	**24**		
**Visit**	**1**	**2**	**3**	**4**	**5**	**6**	**7**		
Informed consent[1]	X								
Randomisation		X							
Medical history/ demographic/ clinical information	X								
Physical examination (complete/dedicated)	X		X	X	X	X	X	X	
AKPS	X	X	X	X	X	X	X	X	
Vital signs	X		X	X	X	X	X	X	
SBT (Short Blessed Test)	X								
12-Lead ECG	X		X	X	X			X	
Chemistry/Hematology	X		X		X		X	X	
CRP		X	X		X		X	X	
IL-6		X	X		X*		X	X	
Urine pregnancy	X	X			X				
FAACT A/CS questionnaire	X	X	X	X	X	X	X	X	
Prior and concomitant medication data	X	X	X	X	X	X	X	X	X
Harms		X	X	X	X	X	X	X	X
Survival (overall and cancer-specific) –date of death		X	X	X	X	X	X	X	X
Height		X							
Body weight (from which BMI will be calculated)		X	X	X	X	X	X	X	
Self-reported weight loss in the last 1, 6, 12 months		X							
BIA (Bio-electrical impedance)		X			X*		X	X	
DEXA scan		X			X*		X	X	
CT scan		X^						X^	
FACIT-F questionnaire		X		X	X		X	X	
GIC (Global Impression of Change)			X	X	X		X	X	
CGI-S (Clincial Global Impression of disease severity)		X	X	X	X	X	X	X	
CGI-I (Clinical Global Impression of improvement/change)			X	X	X	X	X	X	

Assessment	Screening	Baseline	Visit Day Treatment Period			Extension		Exit/ Early Termination (Withdrawal)[a]	Follow-up[b]
**Week**			**4**	**8**	**12**	**18**	**24**		
**Visit**	**1**	**2**	**3**	**4**	**5**	**6**	**7**		
EQ-5D-5L - quality of life		X		X	X		X	X	
ICECAP-A - quality of life[2]		X		X	X		X	X	
SQUASH questionnaire (Physical activity undertaken)		X	X	X	X	X	X	X	
Food diary (review diary)		X	X	X	X	X	X	X	
TUG (Timed *up and go* test)		X	X	X	X	X	X	X	
Step count (review diary)		X	X	X	X	X	X	X	
Acceptability of the study and proxy					X*		X	X	
SCLC treatment regimen and dose or interval adjustments		X	X	X	X	X	X	X	X
Unplanned healthcare contacts (review diary)		X	X	X	X	X	X	X	
Response to cancer therapy for SCLC and adverse events					X		X	X	X
Check compliance (review diary)			X	X	X	X	X	X	
Dispense (D) new and/or return (R) unused medication for study		X (D)			X (R)	X (D)	X (R)	X (R)	

AKPS, Australia-modified Karnofsky Performance Status; BMI, Body Mass Index; ECG, Electrocardiogram; HbA1c, glycated haemoglobin, CPR, C-reactive protein; FAACT, Functional Assessment of Anorexia Cachexia Treatment; DEXA, dual x-ray absorptiometry; CT, computed tomography; FACIT-F, Functional Assessment of Chronic Illness Therapy-Fatigue; SQUASH, Short Questionnaire to Assess Health Enhancing Physical Activity; GIC, Global Impression of Change; CGI-S, Clinical Global Impression of Severity; CGI-I, Clinical Global Impression of Improvement; SCLC, small cell lung cancer. TUG – Timed Up and Go test.

[a] Occurs after the last dose of the study intervention (last day of data collection, or when treatment is ceased, if cessation prior to Week 12 or Week 24 occurs)

[b] First telephone follow-up visit will occur 7 days after the Week 12 or Week 24 (if did not elect to enter extension) visit or the early termination visit due to withdrawal if treatment was ceased early. Subsequent telephone follow-up visits will occur every 7 days thereafter for a period of 4 weeks. There will be 4 follow-up visits in total.

[1]Informed consent will be obtained prior to any study-related screening procedures.

[2]ICECAP-A will be administered after the EQ-5D-5L questionnaire

*Only if people elect NOT to enter the extension.

^ All electronically stored data from chest CT scans whenever they are done clinically will be used for evaluation of change in muscle mass at the thoracic (T4) level.

Table 6-2 Schedule of Study Assessments for caregivers (Screening to Week 12 and optional extension – Weeks 13-24)

**Assessment**	Patient screening	Baseline	Visit Day Treatment Period			Extension	
**Week**			**4**	**8**	**12**	**18**	**24**
**Visit**	**1**	**2**	**3**	**4**	**5**	**6**	**7**
Informed consent[1]	X	X^					
EQ-5D-5L - quality of life		X			X*§		X§
Carer Experience Scale (CES)		X			X*§		X§
Diary (daily records by caregiver)		X	X	X	X	X	X

[1]Informed consent will be obtained prior to any study-related procedures.

^OR (if consent not obtained during visit 1)

*Only if patient elect not to enter the extension.

§ Or withdrawal

7.0 PARTICIPANT TIMELINE

7.1 Visit 1 (Screening)

If the Investigator considers a person to be potentially eligible for the study, written informed consent for participation in the study must be obtained prior to any study-related procedures. The following procedures will be performed, and data collected during this visit for eligibility purposes:

**Clinician**	**Patient-reported outcomes**
Informed consent	FAACT A/CS (score of ≤37)
Demographic and Clinical information (including on the tumour, staging of disease, planned treatment and treatment undertaken to date, etc.)	
Medical history	
Prior and concomitant medication	
Complete and dedicated physical examination	
Vital signs	
Short Blessed Test (SBT score ≥10)	
AKPS Performance Scale (score ≥50)	
12-Lead ECG	
Laboratory blood tests* including random glucose (≤11mmol/L), HbAc1 (≤7%), AST and ALT (≤ 5 x ULN), and renal function (CrCl >20 mL/minute)	
Urine pregnancy test for females of childbearing potential	
Provide participant diary and pedometer – instruct to complete step count and 3-day food diary and bring to baseline visit	
**Caregiver:** Informed consent	

* Ensuring that the study involves minimal burden to participants is crucial in this study, therefore, in some instances (e.g., if clinical picture has not otherwise changed), blood samples taken as part of diagnosis as well as pre-chemo/radiation therapy can be used for screening.

Based on the relevant above mentioned assessments outcome, the Investigator will decide if the person is eligible for the study. If eligible, they will be given the study diary and pedometer (if applicable) to complete the 3-day food diary and step count and bring it to baseline visit (Visit 2). . The study nurse will book DEXA scans at screening visit for participant to have it done as part of baseline assessment.

7.2 Visit 2 (Baseline/ Randomisation)

Participant will arrive at the clinical site where Investigator or designated personnel will re-check the inclusion and exclusion criteria. If person is confirmed eligible, authorised study personnel will proceed with

randomisation procedures. The study kit number to be dispensed to the participant's home will be provided **only after** all the following assessments are completed for baseline measurements.

Baseline assessments can occur at any time between screening and ~3 business day prior to day1 of Cycle 2 of chemotherapy. This is to allow time for the study medication to arrive at the participant's home which should be no later than one day prior to day 1 of Cycle 2 of chemotherapy. The first day a participant takes the study medication is considered Day 1.

The following procedures will be performed, and data collected during this visit:

**Clinician**	**Patient-reported outcomes**
Re-check exclusion criteria	FAACT A/CS
Randomisation	FACIT-F
BIA	EQ-5D-5L
Body weight, height, BMI, self-reported weight in the past 1, 6 and 12 months	ICECAP-A
DEXA scan	SQUASH
TUG test	Daily diary
AKPS	
CGI-S	
Laboratory blood tests* (CRP, IL-6)	
Urine pregnancy test for females of childbearing potential	
Check diary (step count, 3-day food diary, unplanned healthcare contacts)	
Prior and concomitant medication	
Record information on SCLC treatment regimen and any adjustments (dose, interval, protocol, etc.) as relevant	
Review available CT scans	
Toxicity/ AEs	
Survival (date of death)	
Information about medication and delivery at home**	
Reminders: concurrent medications, next visit and keep records in diary and bring it to next visit	
Provide standard physical activity and diet advice leaflets***	
**Caregiver:** Informed consent, EQ-5D-5L, CES, Provide Caregiver Diary	

* Ensuring that the study involves minimal burden to participants is crucial in this study, therefore, in some instances (e.g., if clinical picture has not otherwise changed), blood samples taken as part of diagnosis as well as pre-chemo/radiation therapy can be used for screening.

** The participant will be randomised with the study kit to be delivered to their home within ~3 business days. Study drug should be commenced the next morning after delivery (at least 1 hour prior to first meal of the day) or on the same day of delivery if participant has not eaten first meal of the day. A follow up call will follow baseline Visit to record the date and the precise time (hh:mm) the study drug is taken in the source records as well as on the relevant eCRF page.

*** All participants will receive standard physical activity and diet advice leaflets from the study nurse as part of a multi-modal intervention.

7.3 Visit 3 (Week 4)

The patient will return to the trial site on Visit 3. The following procedures will be performed, and data collected during this visit:

Clinician	Patient-reported outcomes
Vital signs	FAACT A/CS
Complete and dedicated physical examination	SQUASH
Body weight, BMI	GIC
TUG test	Daily diary
AKPS	
CGI-S/ CGI-i	
12-lead ECG	
Laboratory blood tests* (hematology, chemistry, CRP, IL-6)	
Check diary (medication compliance, step count, 3-day food diary, unplanned healthcare contacts)	
Prior and concomitant medication	
Record information on SCLC treatment regimen and any adjustments (dose, interval, protocol, etc.) as relevant.	
Review available CT scans	
Toxicity/ AEs	
Survival (date of death)	
Reminders: concurrent medications, next visit, keep records in diary and bring it to next visit.	

* Ensuring that the study involves minimal burden to participants is crucial in this study, therefore, in some instances (e.g., if clinical picture has not otherwise changed), blood samples taken as part of diagnosis as well as pre-chemo/radiation therapy can be used for screening.

7.4 Visit 4 (Week 8)

The patient will return to the trial site on Visit 4. The following procedures will be performed, and data collected during this visit:

Clinician	Patient-reported outcomes
Vital signs	FAACT A/CS
Complete and dedicated physical examination	FACIT-F
Body weight, BMI	EQ-5D-5L
TUG test	ICECAP-A
AKPS	SQUASH
CGI-S/ CGI-i	GIC
12-lead ECG	Daily diary
Laboratory blood tests* (hematology, chemistry, CRP)	

Check diary (medication compliance, step count, 3-day food diary, unplanned healthcare contacts)	
Prior and concomitant medication	
Record information on SCLC treatment regimen and any adjustments (dose, interval, protocol, etc.) as relevant.	
Review available CT scans	
Toxicity/ AEs	
Survival (date of death)	
Reminders: concurrent medications, next visit, keep records in diary and bring it to next visit.	

* Ensuring that the study involves minimal burden to participants is crucial in this study, therefore, in some instances (e.g., if clinical picture has not otherwise changed), blood samples taken as part of diagnosis as well as pre-chemo/radiation therapy can be used for screening.

7.5 Visit 5 (Week 12) – PRIMARY ENDPOINT

The patient will return to the trial site on Visit 5. Participants will be offered to go into the extension phase of the study. The following procedures will be performed, and data collected during this visit:

**Clinician**	**Patient-reported outcomes**
Vital signs	FAACT A/CS
Completed and dedicated physical examination	FACIT-F
Body weight, BMI	EQ-5D-5L
TUG test	ICECAP-A
AKPS	SQUASH
CGI-S/ CGI-i	GIC
12-lead ECG	Daily diary
Laboratory blood tests* (chemistry, hematology, CRP)	
Check diary (medication compliance, step count, 3-day food diary, unplanned healthcare contacts)	
Prior and concomitant medication	
Record information on SCLC treatment regimen and any adjustments (dose, interval, protocol, etc.) as relevant.	
Review available CT scans	
Toxicity/ AEs	
Survival (date of death)	
**DOESN'T OPT IN to extension phase (End of Treatment)**	
BIA	Acceptability of study tool
DEXA scan	Daily diary - return
Laboratory blood tests* (IL-6)	
**Caregiver:** EQ-5D-5L, CES, Return Caregiver Diary	
**OPT IN to extension phase**	
Urine pregnancy test for females of childbearing potential	
Information about medication and delivery at home	

Reminders: concurrent medications, next visit, keep records in diary and bring it to next visit	

* Ensuring that the study involves minimal burden to participants is crucial in this study, therefore, in some instances (e.g., if clinical picture has not otherwise changed), blood samples taken as part of diagnosis as well as pre-chemo/radiation therapy can be used for screening.

7.6 Visit 6 (Optional Extension Phase - Week 18)

The patient will return to the trial site on Visit 6. The following procedures will be performed, and data collected during this visit:

**Clinician**	**Patient-reported outcomes**
Vital signs	FAACT A/CS
Complete and dedicated physical examination	SQUASH
Body weight, BMI	Daily diary
TUG test	
AKPS	
Check diary (medication compliance, step count, 3-day food diary, unplanned healthcare contacts)	
Prior and concomitant medication	
Record information on SCLC treatment regimen and any adjustments (dose, interval, protocol, etc.) as relevant.	
Review available CT scans	
Toxicity/ AEs	
Survival (date of death)	
Reminders: concurrent medications, next visit, keep records in diary and bring it to next visit.	

7.7 Visit 7 (Optional Extension Phase - Week 24)

The patient will return to the trial site on Visit 7. The following procedures will be performed, and data collected during this visit:

**Clinician**	**Patient-reported outcomes**
Vital signs	FAACT A/CS
Completed and dedicated physical examination	FACIT-F
Body weight, BMI	EQ-5D-5L
TUG test	ICECAP-A
AKPS	SQUASH
CGI-S/ CGI-i	GIC
BIA	Daily diary - return
DEXA scan	Acceptability of study tool
Laboratory blood tests* (chemistry, hematology, CRP, IL-6)	
Check diary (medication compliance, step count, 3-day food diary, unplanned healthcare contacts)	
Prior and concomitant medication	

Record information on SCLC treatment regimen and any adjustments (dose, interval, protocol, etc.) as relevant.	
Review available CT scans	
Toxicity/ AEs	
Survival (date of death)	
**Caregiver:** EQ-5D-5L, CES, Return Caregiver Diary	

7.8 Study visit schedule diagram

Eligibility
- *Informed consent*
- *Screening*

Baseline
- *Baseline assessments*
- *Randomisation 1:1*

Day 1
- *Intervention start*

Week 4
- *Week 1-4 dose*
- *Group 1 VS Group 2*
- *Assessments taken for feasibility, desirability and efficacy*

Week 8
- *Week 4-8 dose*
- *Group 1 VS Group 2*
- *Assessments taken for feasibility, desirability and efficacy*

Week 12
- *Week 8-12 dose*
- *Group 1 VS Group 2*
- *Assessments taken for feasibility, desirability and efficacy*
- *Primary endpoint*

Week 18
- *Optional extension period*
- *Week 13-18 dose*
- *Group 1 VS Group 2*
- *Assessments taken for feasibility, desirability and efficacy*

Week 24
- *Optional extension period*
- *Week 18-24 dose*
- *Group 1 VS Group 2*
- *Assessments taken for feasibility, desirability and efficacy*
- *Intervention cessation*

8.0 STUDY PROCEDURE

8.1 Referrals

All people with a confirmed diagnosis of small cell lung cancer should be referred to the study as soon as possible after diagnosis to enable eligibility screening and baseline assessments to be conducted prior to Cycle 2 of chemotherapy or within three weeks from screening visit, if there is no planned chemotherapy. Therefore, potentially eligible participants will be identified by their own clinicians and referred to the study nurse after discussions with potential participants. The study nurse will ask the consultant in charge for permission to approach potentially eligible participants. This referral will be recorded within both the Data Collection Worksheet and eCRF and the participant's clinical file.

Attendance by the study nurse at key clinics will help to remind clinicians of the study and improve the likelihood of participant identification.

Local and central updates will be supplied to referring teams weekly and local prompts will be developed. These prompts will vary across sites according to the most appropriate form decided by the local teams and may include name-badge lanyard prompts, desk and consulting room office notice board prompts, or clinic posters.

8.2 Participant Informed consent process

Obtaining consent for this study will be a process of information exchange between the study staff, the potential participant and any other person the potential participant believes should be included in the discussion. The participant information sheet will be used as a basis for the discussion, which will cover all procedures, benefits, burdens and side effects expected or possible during the study. The participant will be given opportunity (in time and physical capacity) to consider the study and formulate questions. Any questions will be addressed and answered fully. An actual time period is not specified as this will be determined in part by the participant condition. The study nurses will be trained to determine suitable time for discussion of the study against the participants' needs.

Prior to study commencement, during the site initiation visit, the study nurse, site coordinator and the investigator will be trained in consent procedures for this study, with the opportunity to role play scenarios and develop a consent script to ensure all information is fully covered.

After checking with the clinical team to make sure the participant is feeling well enough to be seen, the Site Investigator or delegated staff member will introduce themselves to the participant and undertake the following:

- Explain the study and provide the participant information sheet. Give the participant time and privacy to read the information;
- Check to confirm participant is interested in participating;
- Obtain informed consent from the participant (please see below for details);

The consent form is completed by the Site Investigator or delegated staff member in accordance with the requirements of the HREC and site regulatory governance requirements. The form is signed and dated by the participant in their own handwriting.

If a potential participant is unable to read and if the legally acceptable representative is unable to read, an Impartial Witness should be present during the entire informed consent discussion. The Impartial Witness signs the Consent Form following:

- The Participant Information Sheet and Consent Form (and any other written material supplied) is read to the potential participant and / or their legally acceptable representative.
- The potential participant (or their legally acceptable representative) consents verbally to participation in the study.
- If able to do so, the potential participant (or their legally acceptable representative) signs and dates the Consent Form.

By signing the consent form, the Impartial Witness attests that the information in the Consent Form and any other written information was accurately explained to, and apparently understood by, the participant or the participant's legally acceptable representative, and that informed consent was freely given by the participant or the participant’s legally acceptable representative. The witness can be any adult who observes the participant signing the consent form, and is able to say that the participant was signing of their own free will, but cannot be the researcher.

Where a witness is not required, but there is space on the consent form for 'Witness Signature' this section is struck through by the study staff with their initials and date, to indicate this section is not required as part of the informed consent process.

The completed consent form is copied (at the time of signing or on return to the study office) and filed:

- one copy is to be given to the participant;
- one copy is to be inserted into the medical file (with research sticker on file if required); and
- the original copy is to be filed in the study file.

8.3 Caregiver consent

Any participant who has consented to join the study and who has been shown to be eligible will be asked to nominate their primary caregiver and for permission to approach them regarding the study/sub-study. The caregiver will then be approached and asked if they would be prepared to complete the caregiver unmet needs questionnaire, diary, and QoL assessments as per the study schedule, as well as participate in the sub-study schedules (detailed separately in the Sub-study Procedures Manual). If in agreement, they will be given the caregiver information sheet and asked to give informed consent for their participation. A primary caregiver should be 18 years and over and is defined as a spouse/relative, partner or friend of the patient who provides care for the person and not someone who has been employed to care for the person. Consent by the caregiver, or otherwise, will not have any effect on the continued participation of the eligible participant.

The completed caregiver consent form is copied (at the time of signing or on return to the study office) and filed:

- one copy is to be given to the caregiver;
- one copy is to be inserted into the medical file (with research sticker on file if required); and
- the original copy is to be filed in study file.

8.4 Screening for eligibility

A Participant Master Index ([PMI] a document that tracks participant names, ID numbers, and progress through the study, developed and maintained at each site) will be kept of all potentially eligible participants including the reasons for non-entry.

Participants suitable for entry who are approached about the study and who have given informed consent will undergo a review of eligibility criteria and complete the eligibility screening:

- Some items will be obtained while in discussion with the potential participant.
- Specific items will require intervention or assessments for the purposes of research and can only be completed after informed consent to participate in the study has been obtained.
  - These items are to be left until it appears that the person is likely to be eligible; and
  - Informed consent is to be obtained prior to conducting the interventional assessments.
- Other items will be completed by referring to the person's clinical file or medical record.
- The plan of management will be checked with treating team (i.e. ensure no planned or likely change in management during the study period).

The completed eligibility screening will be reviewed by the Site Investigator and approval to proceed to randomisation will be obtained. If eligibility is confirmed proceed to baseline assessments and randomisation. A screening log will be kept of all potentially eligible participants including the reasons for non-entry. The screening data will be entered into the online database to enable the Project Officer at the ITCC to monitor eligibility of those enrolled in the study and cross reference with the randomisation process.

8.5 Re-screening

In some cases it is possible that potential participants will need to be re-screened, for example:

- If a person consents to participate, meets the eligibility criteria but there is a delay in starting due to a change in situation (family issues, individual request for attending private matter, etc.);
- If the person previously failed eligibility due to an acute event that has now resolved; or
- Medications have now stabilised.

In these situations, if randomisation has not occurred:

- A new eligibility form is used;
- A new ID number is assigned to the participant;
- The participant is flagged as having been re-screened on both the eCRF and the PMI;
- The eligibility form is completed as if being fully screened, data is not copied from one form to the next, but completed using the current clinical situation as documented within the patient clinical notes.

It is not appropriate to re-screen a person if they have previously failed to meet the eligibility criteria and there have been no further changes or treatments that would now indicate that the patient may be suitable.

8.6 Procedure to request randomisation

The third party pharmacy will be contacted whenever a person is undergoing the screening process to warn them of a potential trial candidate. This will be followed up by a confirmatory call as soon as the person's eligibility has been confirmed.

The randomisation request will take the form of the prescription of the study medications. Upon receipt of the prescription, the pharmacist at the third party pharmacy will then prepare the study medication according to the allocation received from the ITCC Central Randomisation Service The allocation email will be filed in the pharmacy study folder.

The pharmacist will identify the study medications for the participant according to the allocation prescription in the supplied schedule and label the blinded study medication kits providing the details as described in section 4.4.

8.7 Prescription of study drugs

All prescriptions for the use of investigational products for clinical trials must be:

- Completed by a person authorised to do so:
    - the Principal site investigator;
    - sub/co investigators;
    - those medically authorised and delegated on the study to prescribe the specific product under investigation.
- Completed on a hospital prescription form and detail full description of the:
    - participant details;
    - study protocol number;
    - stratification details for disease extent and FAACT score
    - drug (this will be anamorelin or placebo);

- dose (this will specify the dose level/s);
- frequency;
- route.

In this study the prescription will therefore read:
ID No. 044/21 'Anamorelin 100mg / Placebo: swallow one capsule whole each morning at least one hour before food. Please supply 1 study medication kit.'

8.8 Treatment commencement

The study medication will commence on the same day of delivery if participant has not eaten first meal of the day (start Day 1). If a fasting state is not possible on Day 1, participants will start study drug on the next possible day but **no later** than one day prior to planned day 1 of Cycle 2 of chemotherapy. Dosing is thereafter to continue daily, 1 hour prior to the first meal of the day.

8.9 Assessment visits

Participants will visit the study centre at baseline, Weeks 4, 8 and 12 (and 18 and 24 for those in the extension phase). During the visit, the study nurse will take the measures and assessments as outlined in the table of study measures (Table 6-1) and record the visit in the Data Collection Worksheet for that time point. This visit will also be recorded within the participant clinical file. Participants will be offered remuneration to cover parking or taxi fares to attend the study clinical visits, if required.

8.10 End of treatment assessments

Collection of end of treatment data will occur following the last dose of the study intervention (or when treatment is ceased, if earlier cessation occurs). During the visit, the study nurse will take the Week 12 measures and assessments as outlined in Table 6-1 of study measures and record the visit in the Data Collection Worksheet and eCRF. This visit will also be recorded within the participant clinical file, along with any instructions or changes regarding ongoing management.

If early cessation due to an AE, all associated documents will be completed (Serious Adverse Event (SAE) report, AE assessment score etc.).
An AE with a grade of 3 (that has not responded to symptomatic treatment instituted by the treating physician according to local protocols) or 4 will activate treatment cessation and an AE report will need to be completed and submitted. Please refer to Section 10 for further information about AE and SAE reports.

8.11 Withdrawal assessments

If participants are to be withdrawn, a 'Withdrawal Data Collection Worksheet" will be completed by the study nurse on instruction from the investigator. The early termination/withdrawal assessments outlined in Table 6-1 of study measures will be completed [as soon as practicable/within 7 days of withdrawal] and reason for withdrawal will be recorded.

8.12 Follow-up phase assessments

All participants will be followed by their clinician for continuing care, irrespective of the point at which they exit the study. For all study participants, regardless of above choices, study outcomes and collection of data for economic evaluation will occur until the end of 12 weeks after randomisation, unless consent is withdrawn.

Participants will be followed up by telephone weekly irrespective of their place of care for 4 weeks or death or withdrawal of consent, whichever is the shorter period. The date of death will be collected from medical records even if this occurs after the 4-week follow-up. The contact data will be recorded in the follow-up Data Collection Worksheets and eCRF, and will provide data on compliance with cancer treatment, side effects and long term clinical and economic outcomes. The nurse will use the telephone number recorded in the discharge details and will clearly identify themselves. The nurse will ask for information as outlined in the data collection worksheets and will record this while talking with the participant. A medication list will be updated at each contact to record actual prescribed and taken medications since the preceding visit.
Date of death will be recorded for all participants collected from existing medical records.

8.13 Extension phase criteria

At the end of the 12 weeks, participants are invited to enrol in an extension phase where they will continue to receive blinded intervention medication for another 12 weeks. The optional extension (for Weeks 13-24) is entirely based on participant's preference. During the extension phase frequency of AEs, quality of life, medication usage and compliance will be measured up to death or 28 days after ceasing study medication (whichever is the shorter period). These data will be collected via a weekly telephone call to the participant if they have been discharged home and recorded in the follow-up Data Collection Worksheet and eCRF as outlined above. The extension assessments outlined in Table 6-1 of study measures will also be completed.

8.14 Sub-study

The sub-study is detailed in a separate document – the Sub-study Procedures Manual.

9.0 STUDY ADMINISTRATION AND MONITORING

9.1 Reporting of adverse events

All AEs will be reported via an online reporting system to enable study-wide reporting. The ITCC has an SOP for AE reporting that will operate at all study sites (SOP 5.17, Adverse Event Reporting). In addition, there will be specific events and reporting mechanisms required due to the nature of the study drug. This is described below. SAEs will be reported to ITCC, Human Research Ethics Committees (HREC) as per their standard operating procedures; and to the Medical Monitor within 24 hours of knowledge of the event.

9.1.1 The Trial Management Committee

A Trial Management Committee (TMC) will be created for this study. Each meeting of the TMC will receive from the ITCC a summary report of the AEs reported by the investigators, along with data and recruitment updates. Each summary report will be generated from the on-line entry of AE reports by participating sites into REDCap. This summary report will be reviewed for reporting compliance, trends in events, and outstanding events that require specific attention. Minutes will be taken at all Trial Management Committee discussions, with action items detailed, and reviewed at the subsequent meeting. The TMC will not have access to unblinded reports of AEs.

9.1.2 Human Research Ethics Committees

AEs and SAEs are to be reported to site HRECs as per each HREC reporting guidelines and where appropriate the Medical Monitor in the format and timeframe stipulated by each individual committee.

9.1.3 Medical Monitor

A Medical Monitor (MM) will be assigned to review and evaluate information relevant to the safety of the investigational product used. The MM has the responsibility to review and evaluate information relevant to the product safety throughout the implementation of the protocol at all participating sites.

The MM will be responsible for providing safety oversight and reviewing the protocol (e.g., study halting rules) and information about the study product as it becomes available, such as the reportable safety events (SAE's).

The MM, in consultation with the protocol investigator team and the Sponsor will provide safety review during the execution of the clinical trial. This oversight includes reviewing safety information and providing applicable recommendations. The MM will provide recommendations, as appropriate, to members of the study and investigator team. This data and safety review facilitates early detection of safety signals and maximizes the chances for continued appropriateness of the research and protection of human participants.

Based on a synthesis of this information, the MM will provide appropriate recommendations to the Sponsor, participating recruiting sites and approving HRECS.

9.1.4 Adverse events

AEs are defined as any untoward or unexpected occurrence in a patient or clinical investigation participant where the occurrence does not necessarily have a causal relationship with the study intervention.

There are circumstances where AEs will not be reported, however their occurrence will still be recorded in the data collection worksheets and eCRF. Examples are:

- an expected side effect from a study intervention, such as constipation unless the side effect required additional treatment or assessment;
- signs or symptoms associated with the disease or disorder under study, unless they are more severe than expected;
- social admission to hospital.

9.1.5 Serious adverse events

SAEs are any untoward medical occurrence that: meets one or more of the following criteria/outcomes:

- death;
- life-threatening (i.e. at immediate risk of death);
- in-patient hospitalisation or prolongation of existing hospitalisation;
- persistent or significant disability/incapacity;
- congenital anomaly or birth defect;
- other medically relevant condition judged as serious.

- "Life threatening" means that the participant was at immediate risk of death from the event as it occurred. It does not include an event that, had it occurred in a more serious form, might have caused death.
- "Requires inpatient hospitalisation" is defined as hospital admission for treatment of the AE. Hospital admission for scheduled elective surgery would not be an SAE.
- "Other medically relevant condition judged as serious" is where medical and scientific judgment should be exercised in deciding whether expedited reporting is appropriate in other situations, such as important medical events that may not be immediately life-threatening or result in death or hospitalisation but may jeopardise the participant or may require intervention to prevent one of the other outcomes listed in the definition above. These should also usually be considered serious. Examples of such events are intensive treatment in an emergency room or at home for allergic bronchospasm; blood dyscrasias or convulsions that do not result in hospitalisation; or development of drug dependency or drug abuse. A diagnosis of a new cancer during the course of the treatment should be considered as medically important.

Progression of a participant's underlying condition leading to one of the above should always be reported as a serious (but expected) AE, which is unrelated to protocol treatment, or caused by failure of the anticipated therapeutic effect of the study drugs/intervention.

The expected study population have an underlying disease that is expected to significantly shorten life expectancy, they are already termed palliative and are expected to die within a short period of time. In this study, a number of SAEs may be anticipated, but these are unlikely to be attributable to the study intervention.

The conditions recognised as being excluded from the usual SAE reporting timeframes (but are still to be recorded as SAEs) are as follows:

- Where participants are admitted as a planned admission due to respite, family or social issues, or for pre-planned treatment.
- Where participants are admitted (or admission is prolonged) due to a documented expected deterioration in their condition due to the underlying disease process, or where the admission is prolonged for this reason.
- Where participants die due to a well-documented decline in their condition due to the underlying disease process.

In all other cases, SAEs will be reported by the study site, to the ITCC by completing a Serious Adverse Event Report Form and sending it to the ITCC within 24 hours of first knowledge of the event per ITCC SOP 5.17 Adverse Event Reporting. SAEs will be reported according to the requirements of the hospital HREC and/or local Research Governance Office (RGO) and also reported within 24 hours to the MM. A subset of all deaths that occur during the study including during follow-up will be randomly selected for evaluation by the MM.

9.1.6 Criteria for assessing severity

Severity of AEs will be assessed according to Good Clinical Practice (GCP) guidelines and NCI-CTCAE (V5) [51].

9.1.7 Criteria for assessing causality

The site investigator will assess each AE for relatedness or causality of the intervention and the event. A guide to grading the degree of certainty about such a relationship is available at https://www.who.int/medicines/areas/quality_safety/safety_efficacy/WHOcausality_assessment.pdf. A summary of the grading is as follows:

**Unrelated**	Where the AE is clearly not related
**Unlikely**	Where the AE does not have a clear relationship to the intervention
**Possible**	Where the AE follows a known pattern of response
**Probable**	Where the AE reduces or ceases with withdrawal of the intervention
**Definite**	Where the AE ceased with withdrawal of the intervention and recurs with re-exposure.

As this study is blinded, all adverse and serious adverse events should be assessed for causal relationship assuming that the participant has been receiving anamorelin. When determining whether an AE or SAE is expected or not, please refer to the reference safety information provided in the Investigator Brochure. If the event is listed in the Investigator Brochure or specified as a symptom of special interest in section 9.1.9, the AE or SAE should be assumed to be ‘Possible’, ‘Probable’ or ‘Definite’ unless there is a clear alternative cause.

Definite/Certain: a clinical event, including laboratory test abnormality, occurring in a plausible time relationship to drug administration, and which cannot be explained by concurrent disease or other drugs or

chemicals. The response to withdrawal of the drug (dechallenge) should be clinically plausible. The event must be definitive pharmacologically or phenomenologically, using a satisfactory rechallenge procedure if necessary.

Probable/Likely: a clinical event, including laboratory test abnormality, with a reasonable time sequence to administration of the drug, unlikely to be attributed to concurrent disease or other drugs or chemicals, and which follows a clinically reasonable response on withdrawal (dechallenge). Rechallenge information is not required to fulfil this definition.

Possible: a clinical event, including laboratory test abnormality, with a reasonable time sequence to administrations of the drug, but which could also be explained by concurrent disease or other drugs or chemicals. Information on drug withdrawal may be lacking or unclear.

Unlikely: a clinical event, including laboratory test abnormality, with a temporal relationship to drug administration which makes a causal relationship improbable, and in which other drugs, chemicals or underlying disease provide plausible explanations.

Conditional/Unclassified: a clinical event, including laboratory test abnormality, reported as an adverse reaction, about which more data is essential for a proper assessment, or the additional data is under examination.

Unassessable/Unclassifiable: a report suggesting an adverse reaction which cannot be judged because information is insufficient or contradictory, and which cannot be supplemented or verified.

9.1.8 Time period for assessing AEs and SAEs

At each visit, the participants are encouraged to mention any problems since the last visit. In addition, the following standard questions should be asked:

- Have you had any medical problems since the last visit or study assessment?
- Have you started any new medications, other than given to you in this study, since the last visit or assessment?

For all randomised participants: all AEs, irrespective of causality, will be assessed and recorded as per ITCC SOP 5.17 Adverse Event Reporting. The time period includes the time from screening, the 12-week treatment period/the 4-week extension phase and the 4-week follow-up period (by telephone).

For patients who meet the eligibility criteria and then do not proceed to randomisation, any AEs detected during that window of time, should also be reported. If an AE is the reason that a person does not proceed to randomisation, this should be recorded within the eCRF, AE report and in the clinical record, irrespective of causality or seriousness.

9.1.9 National Cancer Institute, Common Terminology Criteria for Adverse Events. (NCI CTC AE).V5.0

These criteria have been used to determine AEs likely to occur during the study period and will be used to determine AE reporting and study progress. Criteria specific to the expected events know to be associated with anamorelin have been listed. This is administered by study staff.

Symptoms will be identified during each visit using criteria established by the National Cancer Institute, and will be graded accordingly. Specifically, for this study, the symptoms of interest will be:

- Symptomatic hyperglycaemia (dizziness, cold sweat, loss of consciousness, abdominal pain, shortness of breath, confusion), constipation, nausea, vomiting.
- Blood glucose increased (investigation)
- Symptoms of cardiac dysfunction (PR prolongation and increased QT interval measured on ECG), blurred vision, breathlessness, chest pain, syncope, palpitations
- Headache
- Peripheral oedema

A grade of 3 (that has not responded to symptomatic treatment instituted by the treating physician according to local protocols) or 4 will activate cessation of study intervention and an SAE report. All AEs will be collated by the project officer and reported to the executive committee on a monthly basis. AE rates will form part of the Key Performance Indicators for the study and will be reported on a regular basis to the Trial Steering Committee and the ethical review boards of the participating sites.

9.1.10 Adverse Event Assessment Diagram

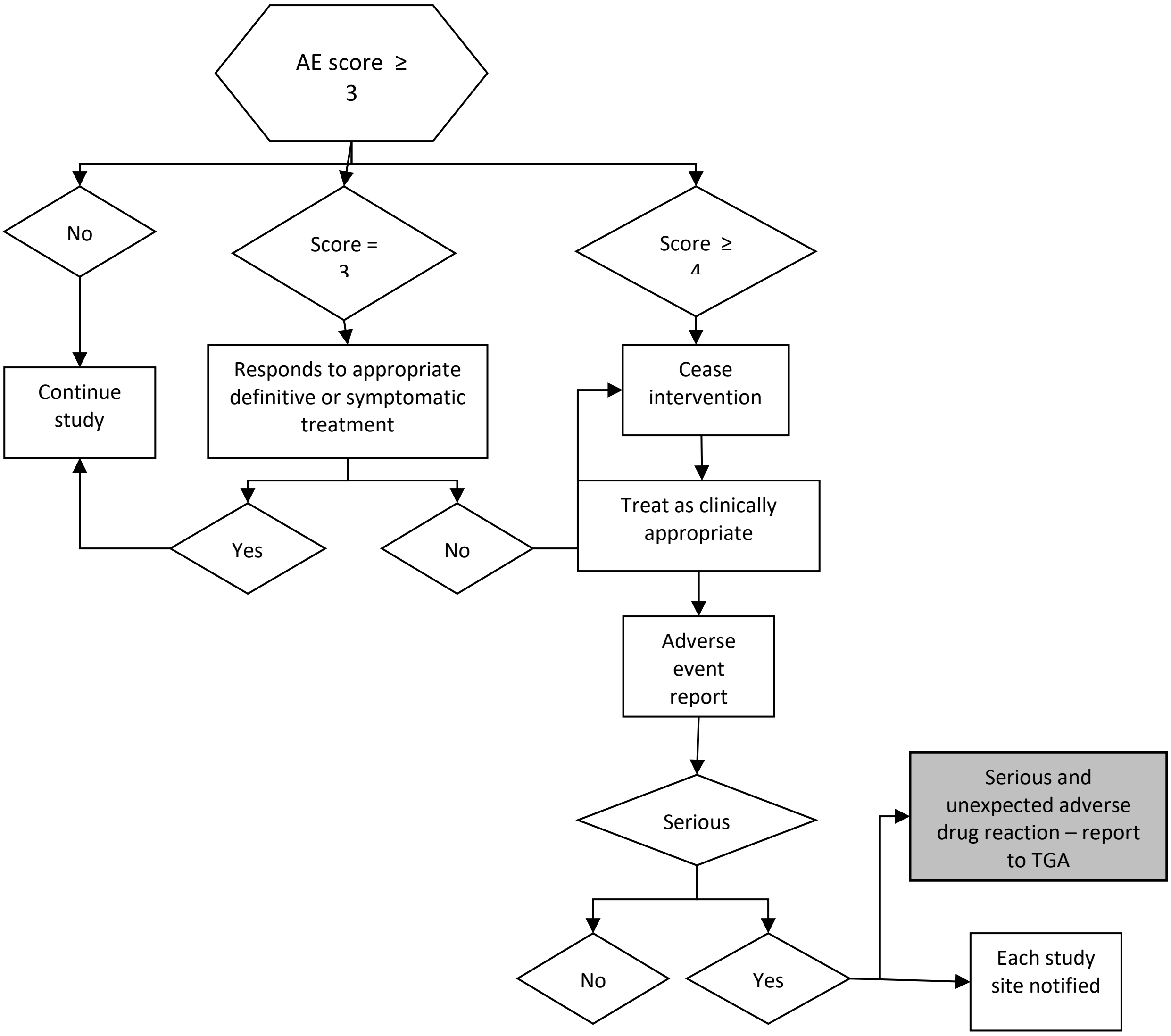

The ITCC will distribute the SAEs to the relevant parties and committees per ITCC SOP 5.17 Adverse Event Reporting and in accordance with state and territory guidelines, the requirements of the approving central Ethics Committee and local governance offices and the Medical Monitor. In addition, events deemed to be both serious and unexpected, and related to the study intervention, will be reported by the ITCC to the Therapeutic Goods Administration (TGA) via their website (https://aems.tga.gov.au/). The online reporting is to be used to enable reporting between sites, local forms of reporting are also to be used for reporting to local sites.

9.1.11 Follow-up of AEs and SAEs

After the initial report, investigators are required to follow-up each AE and provide further information both to the ITCC and the hospital HREC and/or RGO. All events reported as ongoing are to be reviewed at subsequent visits or appointments in order to report progress and resolution. All events are to be followed until:

- resolution;
- the condition of the participant stabilises;
- the event can be explained;
- the participant is lost to follow-up; or
- death.

Reports are to contain details of follow-up investigations, result reports or reports from other consultations, and are to be updated in a follow-up report to the ITCC and the approving HREC and/or RGO.

9.1.12 Post study AEs and SAEs

A post study event is defined as any event that occurs outside of the time period described in section 9.1.8 of the protocol. Investigators are required to report any events they become aware of if:

- the event occurs at any time after study participation has ceased; and
- the event is assessed as being reasonably related to the study intervention.

Investigators are not obliged to actively seek events that occur after the study period defined in 9.1.8. Date of death will be required for all participants regardless of when they withdrew from the study up to the dataset being closed.

9.2 Auditing/Monitoring

9.2.1 Peer review and site visits

Each study site will be reviewed by the ITCC Project Officer (or delegate) prior to recruitment commencement, when the site coordinator and study nurse will be assessed as appropriate, and trained in the data collection, data entry, and filing and other trial procedures in order to comply with Good Clinical Practice (Site Initiation

Visit, SIV). The assessment will be recorded and a copy sent to the study site. Ongoing peer review will be undertaken via regular study nurse telephone links and ongoing assessment by the study investigator.

9.2.2 Pharmacy training

At the site initiation visit, the third party pharmacy will be visited or telephoned by the National Project Officer (ITCC)/delegate. At this time, the pharmacy staff will be trained on the pharmacy procedures, the protocol will be reviewed in detail and a pharmacy manual provided. The manual has been prepared with the input and advice of experienced trial pharmacists prior to finalisation.

9.2.3 Monitoring visits

Internal monitoring of the study is described in detail in the Monitoring SOP (5.18, Monitoring).

Briefly, each study site will be monitored by staff from the ITCC at initiation, mid-recruitment and study closure where all study procedures, recording, reporting and maintenance will be checked, including the pharmacy records. This will include data quality, protocol violations, AE reporting, participant existence and eligibility, and other aspects to determine Good Clinical Practice compliance. Monitoring for this study will take a pragmatic and risk-based approach, where on-site visits will be undertaken based on the outcomes of continuous central remote monitoring of recruitment, data quality, compliance, protocol violations and direct communications.

9.3 Quality control

9.3.1 Training procedures:

The following training procedures will be conducted to ensure quality control. Competency will be recorded at the ITCC with a copy of the training records filed at each study site.

**Person trained**	**Description**	**Assessed by**
All site staff	ICH GCP training	National manager (PaCCSC/CST)
Study nurse	Blood sampling	Pathology department
Study nurse	Eligibility assessment	Site investigator
Study nurse	Consent procedure	Study coordinator
Investigator,<br>sub investigators	ICH GCP training; Protocol	National manager (PaCCSC/CST)<br>Chief investigator
Study nurse, investigators	Data management	National manager (PaCCSC/CST)

Person trained	Description	Assessed by
Medical staff	Prescription	Site investigator
Clinical trials pharmacist	Randomisation, medication preparation procedures	National manager (PaCCSC/CST)<br>Chief investigator
Investigator, sub investigators	Safety assessments	National manager (PaCCSC/CST)<br>Chief investigator

9.3.2 Blood collection

Venous blood samples will be drawn for eligibility screening, baseline and follow-up assessments. Ensuring that the study involves minimal burden to participants is crucial in this study, therefore, in some instances (e.g., if clinical picture has not otherwise changed), blood samples taken as part of diagnosis as well as pre-chemotherapy/radiation therapy can be used for screening. The results will be held in the participant study file as source data. This will be collected as per the site's SOP for venepuncture, with results reviewed and signed off by the Site Investigator or delegated medically trained staff member (such as sub-Investigator).

Each study site will keep a copy of the pathology service guidelines for obtaining, transporting and storing blood samples.

9.4 Data Management

9.4.1 Data handling and record keeping

The ITCC has a number of Standard Operating Procedure that will apply to all sites for the management of study data. Specifically, the following Standard Operating Procedures (SOPs) apply:

- SOP 5.5.1 Electronic Data Handling
- SOP 5.23.2 CRF completion
- SOP 8.0 Essential documents
- SOP 8.4.1 Archiving of research/project materials
- SOP 8.42 Record destruction

9.4.2 Direct access to source data

A statement of permission to access source data including medical, health and mental health records for regulatory and audit purposes is included within the participant consent form with explicit explanation about this given as part of the consent process. Specifically, access will be required by study staff (including investigators, site coordinators and study nurses), Human Research Ethics Committees (HRECs), Medical Monitor and the ITCC data management team. Increasingly, there is a requirement to make the data available at publication dependent on the journal requirements and this would be done in a summarised and de-identified format. So de-identified data may be made available to bona fide researchers for meta-analysis and where requested by journals for publication purposes. Regulatory authorities may require use of the data for subsidy applications. In addition, the aggregated study data will be used to inform the next phase of the study. Any future use of the study data would be at the discretion of the Sponsor/data custodian.

The Data Collection Worksheets listed below will be provided for data entry into the electronic Case Report Forms (eCRF):

- Eligibility
- Baseline
- Intervention
- Treatment Cessation
- Withdrawal
- Follow-up

- Medical Assessment and Screening Form

9.4.3 Data collection

Data will be sourced from the following:

Measure	Source	Completed by:
General demographic details	Clinical file	Study nurse
General medical information and Charlson comorbidity	Clinical file/ medical assessment	Medical officer/ investigator
Concurrent medications	Clinical file	Medical officer
Pathology results	Pathology report	Pathology service
Vital signs	Clinical file	Study nurse
Patient reported outcomes	Questionnaires	Participant
Patient-reported symptom measures;	Worksheet	Study nurse
Physical examination	Worksheet	Medical officer/ Study nurse
Physiological measures: weight, BMI, BIA, DEXA and CT scans for body composition, ECG	Worksheet/record of BIA, DEXA and CT scans, ECG	Study nurse
Adherence: medication, physical activity and dietary advice	Participant diary	Participant
Adverse events, safety	Clinical file and Worksheet	Study nurse

To assist sites in ensuring that the study data collected are complete and consistent, ITCC develops and provides study specific data collection worksheets for use. The worksheets are designed to capture the required data and to enable smooth data entry in the eCRFs through similar question and response structures, where required data fields are highlighted in order to minimise data entry errors and prevent missing data.

Study data including data collected on paper data collection worksheets will be entered and stored on an electronic database. This web-based data capture system enables the application of data validation rules to ensure that required data is entered or that an annotation is provided to explain any missing data. Validation rules to assist with data checking such as rules that apply to expected visit dates or values that fall within a specified range are also used to flag potential data entry errors in real-time and require an annotation to explain entry of erroneous values. The completion of specific system and data entry training is mandatory for all site staff requiring access to the data capture system as per ITCC SOP 5.5.1 Electronic Data Handling.

9.4.4 Electronic recording

Study data will be recorded in a number of files for both the administration of the study and collection of participant data.

- A master index (PMI) will contain confidential participant contact information and will be the only link between individual participants and the ID number. This will be an Excel spreadsheet (Master patient index.xls).

- The Data file will be held and administered in the ITCC and will contain all the participant data as downloaded from the eCRFs. This data will then be transferred to the data set for analysis.

9.4.5 Data entry

Study data will be collected and stored on paper Data Collection Worksheets and then entered onto and managed on REDCap (Research Electronic Data Capture) database or eCRF. The eCRF database will contain the responses to the Data Collection Worksheets, telephone monitoring data and the participant medication use.

Audio data, identified only by study ID will be collected on a digital recording medium and stored temporarily at any of the study or sponsor sites until uploaded to the REDCap database. The original file will then be destroyed.

REDCap is a secure web-based application designed to support data capture for research studies, providing user-friendly web-based case report forms, real-time data entry validation, audit trails and non-identified data export mechanism to common statistical packages. All web-based information transmission in REDCap are protected via Secure Sockets Layer (SSL) encryption (data entry, survey submission, web browsing, etc).

The creator of a REDCap project database may grant other users access to the project and limit those users' privileges as desired. All users are given individual system usernames and passwords. Passwords are at least 9 characters long and have to be alphanumeric. REDCap has an audit trail for all data entry and subsequent alterations to data.

9.4.6 Data querying

Data will be checked according to ITCC Standard Operating Procedure 5.5.1 Data Handling. eCRF data entry will be checked by a series of programmed checks and listing reviews incorporated into the REDCap interface using validation rules. Queries will be either automatically generated or manually added to the eCRF for resolution by the site. Responses to queries will be entered directly into the eCRF by site staff. RedCAP maintains an audit trail of all queries, responses and data corrections. Quality control audits of all key safety and efficacy data in the database are made prior to locking the database.

9.4.7 Data storage

All data collected at each site for each participant will be kept in a participant file (identified by ID number only) which will contain the Data Collection Worksheets, any corrected and amended data, copies of AE reports, file notes, consent forms, pathology reports, medication chart etc. All participant files will be securely stored at each study site in a locked area that is only accessible to study staff and away from the administrative files for the study. All scanned CRFs sent to the Trial Coordination Unit will be filed in a password protected network folder, until study archive. All study files will be stored for 15 years.

If it is the site's policy, identifiable data (consent forms, pathology reports, etc.) may be de-identified and filed with the study documents or alternatively can be stored separately from the participant's study file during the recruitment period to further avoid potential re-identification of participants. All participant files will be reconciled and stored along with all study materials – both hard copy and electronic – consistent with ICH GCP R2 and applicable regulations regarding the retention and disposal of participant records.

10.0 STATISTICS

10.1 Power and sample size

A total of 50 participants with small cell lung cancer will be randomised 1:1 to anamorelin HCl 100 mg or placebo in order to have data at 12 weeks on 40 participants (approximately 20 in each treatment arm). Since this is a feasibility study, it is not required to calculate a fully justified sample size for establishing the efficacy of the intervention.[52] It has been suggested, however, that in feasibility studies, a sample size between 24 and 50 is acceptable.[53, 54] The findings from this study will be used to inform the calculation of a sample size for a future larger scale phase III trial.

Participants discontinued after randomisation will not be replaced.

10.2 Alternative hypotheses to be tested

Feasibility

There will be adequate recruitment and retention of participants with SCLC and anorexia for the phase II trial.

Desirability

Anamorelin will show sufficient clinical signal of difference between the treatment and control arms with acceptable safety measures.

10.3 Analysis of primary outcomes

Feasibility

Primary analysis will be concentrated on the feasibility metrics based on defined thresholds. As this is a feasibility trial, there will be no imputation. Data relating to quantitative variables will be analysed with simple frequencies. Comparison between the intervention and control groups will be compared using appropriate statistical approaches, depending on the nature of the data.

Efficacy outcomes

Changes from baseline to 12 weeks or 24 weeks in each of the secondary endpoints will be calculated. An exploratory analysis of the potential differences between arms will be performed using t-test for continuous normally distributed data or Wilcoxon Test for continuous non-normally distributed data.

10.4 Safety outcomes

All safety and tolerability data will be summarised descriptively. Predictors of harms will be sought in order to inform refinement of the phase III study eligibility. Descriptive statistics will be produced for each of the outcome measures to evaluate the appropriateness of the measures for inclusion in a definitive trial.

10.5 Economic analysis

The feasibility of collecting data for a comprehensive cost effectiveness analysis of anamorelin in people with SCLC and anorexia will be determined in any subsequent phase III clinical trial.

11.0 ETHICS AND DISSEMINATION

11.1 Benefit anticipated from the study

At present, there is no medicine specifically approved for the treatment of anorexia in Australia. This includes no medication registered for the treatment of anorexia in SCLC, despite this condition being relatively common, and the cause of considerable distress to patients, caregivers and clinical staff.

This study therefore proposes to validate the use of a treatment that could provide significant improvement in anorexia where no current therapy exists. The results of this study will provide evidence to conduct a fully powered phase III study into the use of anamorelin for anorexia in people with SCLC. This further study will have the potential, if positive, to provide information to enable this medication to be submitted for approval by the TGA and for listing on the Pharmaceutical Benefits Scheme (PBS) for use in the community. If this further study is negative, given this is an adequately powered study, the results will help to inform clinical practice.

11.2 Assessment of burden and stress

Each of the study measures have been carefully selected to ensure they provide the best possible data with the least impact on the participant and have been validated. As much as possible, the study measures are non-invasive in order to minimise physical stress.

Participants will be asked to provide a blood sample for eligibility confirmation, baseline and follow-up measures. The taking of blood is uncomfortable, but short term. This is the only invasive procedure during the study period and has been kept to an absolute minimum.

Any harms will be carefully monitored to ensure any anticipated discomfort is detected and treated in a timely manner.

Some participants may experience stress associated with completing some of the study measures. This is a vulnerable population, where sensitive issues about ability to continue to function, quality of life and other questions may raise broader issues of psychological distress.

Emotional distress caused by any of the questions in the quality of life measures will be dealt with by members of the palliative care team who would be involved either directly or in consultation with the care of the patients under those circumstances. Although there may be acute distress, the weight of evidence is that such ‘prompt’ questions are in fact an avenue to open up discussions which are well regarded by patients despite their initial potential distress on occasion.

There will be no deception of participants at any stage of the project. Each participant interaction will be undertaken by carefully selected and trained study staff. This training will initially be undertaken in conjunction with investigators and senior research personnel, who have been trained in Good Clinical Practice, to ensure that staff are able to detect and monitor participant distress. Ongoing site monitoring will provide ongoing training opportunities.

The nature of doctor-participant relationships dictates that participants may feel that they are in the dependent position. The investigators and their designees will work to minimise any concern of inappropriate influence—the presentation of the study will be as unbiased as possible, the information sheet and consent forms will be clear, and participants will be able to withdraw from the study at any time. A research team member not directly involved in the clinical care of the participant will obtain consent. Each site will employ a recruitment nurse who will approach participants for permission to present the study. Each recruitment nurse will be trained in presenting the study in such a way that clinical care is separated.

11.3 Potential Risks

11.3.1 Participants of non-English speaking background

The study will include participants whose English is not their first language, as long as they are able to fully understand the study, provide informed consent and complete the assessments/interviews (refer to inclusion and exclusion criteria in section 3.4.2). The research team will assess each participant's level of understanding either by direct conversation or by exposing the participant to some of the questionnaires used in the trial. Research teams participating in this trial include highly experienced clinicians who are used to address cultural differences. There are no expected implications for particular cultural groups coming from this intervention.

11.3.2 Non-participation and withdrawal from the study

Participants will not suffer discrimination if they decline to participate or withdraw from the study. Decision to participate in this study is entirely voluntary and refusal to participate will involve no penalty or loss of benefits to which the participant would otherwise be entitled. In addition, the participants may withdraw from the study at any time without penalty or loss of benefits. Participants' medical care will not be affected by the decision to withdraw from the study. This information will be clearly communicated to participants in the PICF. Participants will continue to be under the clinical care of their referring physician. Non-participation and/or withdrawal from participation will result in continuation of the current clinical care, without consequences. Enrolment into the study by non-clinical care providers will ensure that there can be no influence on clinical decision making.

11.3.3 Use of an unapproved medicinal product/novel intervention:

The interventions used in the active treatment arm of the study include anamorelin HCl which is not currently approved by the TGA in Australia for the treatment of anorexia. There is still limited evidence related to the efficacy and safety profile of anamorelin in patients with anorexia. The current safety profile of this medication was established from a series of Phase II and Phase III clinical trials in patients with advanced lung cancer. The observed side effects and contraindications associated with anamorelin HCl are outlined in the Product Information (Table 4-1). Anamorelin may cause side effects including hyperglycaemia and grade 1 or 2 nausea. Side effects and adverse events of special interest (refer to section 9.1.9) will be carefully monitored to ensure any anticipated discomfort is detected and treated in a timely manner. Recently, the use of anamorelin for improvement of weight loss and anorexia in cancer cachexia in people with NSCLC, gastric cancer, pancreatic cancer and colorectal cancer was approved in Japan for the first time in the world.[16]

11.3.4 Risks associated with dose administration

Participants may be inpatients admitted to the oncology/respiratory/palliative care ward or outpatients. For participants who are inpatients, the study medication will be stored according to the local hospital practice and dispensing and dosing recorded in the medication chart. As dosing administration will be managed by the ward nurses, there is a low risk that medication errors could occur. Ward staff will be trained in the dosing schedule and protocol requirements for treatment cessation.

Participants who are outpatients will be provided the medicine packs at home and will be required to self-administer study medication as prescribed and record the daily dose within their daily diary. There is a moderate risk associated with self-administration of the study medication and medication errors may occur. To minimize the likelihood of medication errors, participants will be trained on dosing requirements of the study and how to store their study medication safely at home prior to every dispensing. Participants will be informed that the study medication must not be removed from its packaging unless it is prior to taking the required dose and must store the medicine packs in a secure location out of reach of children when not required such as in a cabinet and at room temperature below 25◦C. Participants will be informed that if a dose is missed and it is almost time for the next dose (within 16 hours), they should not take the missed dose and should just take the next scheduled dose. If the missed dose was less than 8 hours earlier, then the missed dose may be taken as soon as the participant remembers. In the event of an overdose, participants must contact their doctor, the Poisons Information Centre or go to Accident and Emergency at the nearest hospital. This information will be explained in the patient information and consent sheet and a dosing guide will be provided. The daily diary will be also reviewed at each study visit/telephone contact to check dosing compliance.

11.3.5 Risks associated with study procedures- DEXA scans

As part of this study, participants will be required to undergo DEXA scans at Visit 2, Visit 5 or Visit 7 or withdrawal to track changes in weight and associated changes in fat and muscle mass. Whilst the scans only take 25 minutes to complete, this is additional imaging and radiation exposure than would be typically required as part of routine standard of care. However, the DEXA scan is very safe for most people as it uses very low level of x-ray energy. As part of everyday living, everyone is exposed to naturally occurring background radiation and receives a dose of about 2 millisievert (mSv) each year. The effective radiation dose from the extra DEXA scans required as part of this study is estimated to be less than 0.07 mSv. At this dose level, no harmful effects of radiation have been demonstrated as any effect is too small to measure. The risk is believed to be minimal. Participants will be advised of the minimal risks associated with the DEXA scans in the PICF.

11.4 Confidentiality

11.4.1 Separation of research and clinical responsibilities

There are many distressing symptoms faced by participants with a life limiting illness and there is very little research to support many of the interventions that palliative medicine doctors provide daily around the world.

Although research in this area poses its own unique dilemmas, the ethics of not conducting research into the best management of the dying participants is untenable. Importantly, participants will be cared for as individuals with specific needs; the needs of research will come second. Research staff, (medical or nursing), will clearly identify themselves and the purpose of their visit at their contact with the participant as being part of the research process. Training at the site initiation visit will provide an opportunity for study staff to determine appropriate ways of dealing with clinical situations that might arise during their research visits.

11.4.2 Protection of privacy and preservation of confidentiality

The participants will be allocated a unique ID number. The master list linking identifying participant information and ID number will be maintained in a secure location, separate from the participant files and study database. Data collection worksheet and eCRF tracking will be via participant ID number only. There will be master lists held at each participating site. Study data will be stored and managed on an independent REDCap database instance hosted by the University of Technology, Sydney (UTS). REDCap is a secure and password-protected web-based application where all web-based information transmission is encrypted and data is stored on a local UTS server with local management and support. All study data will be analysed by ID number only.

11.4.3 Use of personal information

Only enough personal information to give a general demographic and disease profile of the participant will be collected. The participant responses collected are limited to those that will address the study's primary and secondary aims.

11.4.4 Retention and Disposal of personal information

Records from the study will be maintained for 15 years after study completion in secure archiving facilities. Site records will be maintained on site using the local archiving arrangements and in line with state regulations for destruction. Once the 15-year waiting period is complete, the data will be erased from the REDCap database server, electronic files will be deleted from password-protected network drives and all paper files will be shredded, including the master list linking participant name and treatment number.

The data will be retained in accordance with good clinical practice recommended by the NHMRC National Statement and the GCP guidelines, and in a form that is at least as secure as the sources from which it was obtained.

11.5 Method and nature of recruitment and advertising

Participants will be recruited on admission to the cancer service and during initial screening in the participating clinics at each site. Advertising brochures will not be provided, only the information sheet and a verbal explanation. Any participant who is approached to take part in this study has the right of refusal. Refusal to take part in this study will not adversely affect the provision or quality of care provided to any participant in any way.

11.6 Access to data

Data use will be restricted according to each individual's role in the study. Study investigators will have access to data by ID number only for the purposes of data monitoring and analysis. The National Project Officer and monitors will have access to all study data, including source data, data collection worksheets, eCRFs, study files and participant medical records for the purposes of data checking, monitoring and preparation for analysis. Study project officers and site coordinators will have access to the local site data collection worksheets/eCRF and the data contained within for the purposes of data collection, data entry and data query resolution. The Medical Monitor will have access to de-identified data for safety and efficacy assessments. Study auditors will have access to data collection worksheets (by ID number only), eCRFs and study files in order to audit the study. Site research ethics committees will have access to local data for audit purposes.

11.7 Sponsor and collaborations

This study is sponsored by the University of Technology Sydney and coordinated by the IMPACCT Trials Coordination Centre (ITCC).

11.8 Dissemination policy

Any requirements for data sharing during the process of publishing results in a peer-reviewed publication will comply with all of the data privacy and Human Research Ethics Committee requirements. Data for the whole study will be held by ITCC for the required period of 15 years.

11.9 Authorship

Publications reporting results from the study will be submitted to international peer review medical journals in accordance with ITCC SOPs. All proposals for publications will be subject to publication protocols that should be submitted to ITCC National Manager for approval in order to avoid controversies, agree on relevant authorship and duplicate publications.

12.0 REFERENCES

1. Zhang, F., et al., *The management strategies of cancer-associated anorexia: a critical appraisal of systematic reviews.* BMC Complement Altern Med, 2018. **18**(1): p. 236.
2. Trajkovic-Vidakovic, M., et al., *Symptoms tell it all: a systematic review of the value of symptom assessment to predict survival in advanced cancer patients.* Crit Rev Oncol Hematol, 2012. **84**(1): p. 130-48.
3. Tarricone, R., et al., *Impact of cancer anorexia-cachexia syndrome on health-related quality of life and resource utilisation: A systematic review.* Crit Rev Oncol Hematol, 2016. **99**: p. 49-62.
4. Vergara, N., et al., *Quality of life and nutritional status among cancer patients on chemotherapy.* Oman medical journal, 2013. **28**(4): p. 270-274.
5. Rodriguez, A.M., E.M. Duus, and J. Friend, *Experience of weight loss and its burden in patients with non-small cell lung cancer: Results of an online survey.* 2016. **34**(26_suppl): p. 71-71.
6. Fitzmaurice, C., et al., *The Global Burden of Cancer 2013.* JAMA Oncol, 2015. **1**(4): p. 505-27.
7. Ross, P.J., et al., *Do patients with weight loss have a worse outcome when undergoing chemotherapy for lung cancers?* Br J Cancer, 2004. **90**(10): p. 1905-11.
8. Yang, R., et al., *Obesity and Weight Loss at Presentation of Lung Cancer are Associated with Opposite Effects on Survival.* Journal of Surgical Research, 2011. **170**(1): p. e75-e83.
9. Tranmer, J.E., et al., *Measuring the Symptom Experience of Seriously Ill Cancer and Noncancer Hospitalized Patients Near the End of Life with the Memorial Symptom Assessment Scale.* Journal of Pain and Symptom Management, 2003. **25**(5): p. 420-429.
10. Anker, M.S., et al., *Orphan disease status of cancer cachexia in the USA and in the European Union: a systematic review.* J Cachexia Sarcopenia Muscle, 2019. **10**(1): p. 22-34.
11. Lindsey, A.M. and B.F. Piper, *Anorexia and weight loss: Indicators of cachexia in small cell lung cancer.* Nutrition and Cancer, 1985. **7**(1-2): p. 65-76.
12. Currow, D.C. and A.P. Abernethy, *Anamorelin hydrochloride in the treatment of cancer anorexia-cachexia syndrome.* Future Oncol, 2014. **10**(5): p. 789-802.
13. Temel, J.S., et al., *Anamorelin in patients with non-small-cell lung cancer and cachexia (ROMANA 1 and ROMANA 2): results from two randomised, double-blind, phase 3 trials.* The Lancet Oncology, 2016. **17**(4): p. 519-531.
14. Currow, D., et al., *ROMANA 3: a phase 3 safety extension study of anamorelin in advanced non-small-cell lung cancer (NSCLC) patients with cachexia.* Ann Oncol, 2017. **28**(8): p. 1949-1956.
15. Aversa, Z., P. Costelli, and M. Muscaritoli, *Cancer-induced muscle wasting: latest findings in prevention and treatment.* Ther Adv Med Oncol, 2017. **9**(5): p. 369-382.
16. Wakabayashi, H., H. Arai, and A. Inui, *The regulatory approval of anamorelin for treatment of cachexia in patients with non-small cell lung cancer, gastric cancer, pancreatic cancer, and colorectal cancer in Japan: facts and numbers.* Journal of Cachexia, Sarcopenia and Muscle, 2021. **12**(1): p. 14-16.
17. Prommer, E., *Oncology Update: Anamorelin.* Palliat Care, 2017. **10**: p. 1178224217726336.
18. Helsinn Healthcare, *Anamorelin HCL Investigator's Brochure*. 2018.
19. Eisenhauer, E.A., et al., *New response evaluation criteria in solid tumours: revised RECIST guideline (version 1.1).* Eur J Cancer, 2009. **45**(2): p. 228-47.
20. Vickers, A.J. and D.G. Altman, *Statistics notes: Analysing controlled trials with baseline and follow up measurements.* BMJ, 2001. **323**(7321): p. 1123-4.
21. Levey, A.S., et al., *A more accurate method to estimate glomerular filtration rate from serum creatinine: a new prediction equation.* Annals of internal medicine, 1999. **130**(6): p. 461-470.
22. Temel, J.S., et al., *Anamorelin in patients with non-small-cell lung cancer and cachexia (ROMANA 1 and ROMANA 2): results from two randomised, double-blind, phase 3 trials.* Lancet Oncol, 2016. **17**(4): p. 519-531.

23. Charlson, M.E., et al., *A new method of classifying prognostic comorbidity in longitudinal studies: development and validation.* J Chronic Dis, 1987. **40**(5): p. 373-83.
24. De Groot, V., et al., *How to measure comorbidity: a critical review of available methods.* Journal of clinical epidemiology, 2003. **56**(3): p. 221-229.
25. Abernethy, A.P., et al., *The Australia-modified Karnofsky Performance Status (AKPS) scale: a revised scale for contemporary palliative care clinical practice [ISRCTN81117481].* BMC Palliat Care, 2005. **4**: p. 7.
26. Bohannon, R.W., *Reference values for the timed up and go test: a descriptive meta-analysis.* J Geriatr Phys Ther, 2006. **29**(2): p. 64-8.
27. Blauwhoff-Buskermolen, S., et al., *The assessment of anorexia in patients with cancer: cut-off values for the FAACT-A/CS and the VAS for appetite.* Supportive care in cancer : official journal of the Multinational Association of Supportive Care in Cancer, 2016. **24**(2): p. 661-666.
28. Farrar, J.T., et al., *Clinical importance of changes in chronic pain intensity measured on an 11-point numerical pain rating scale.* Pain, 2001. **94**(2): p. 149-158.
29. Busner, J. and S.D. Targum, *The clinical global impressions scale: applying a research tool in clinical practice.* Psychiatry (Edgmont), 2007. **4**(7): p. 28-37.
30. Herdman, M., et al., *Development and preliminary testing of the new five-level version of EQ-5D (EQ-5D-5L).* Qual Life Res, 2011. **20**(10): p. 1727-36.
31. Fermont, J.M., et al., *The EQ-5D-5L is a valid approach to measure health related quality of life in patients undergoing bariatric surgery.* PLoS One, 2017. **12**(12): p. e0189190.
32. Walter, D., et al., *Higher quality of life after metal stent placement compared with plastic stent placement for malignant extrahepatic bile duct obstruction: a randomized controlled trial.* Eur J Gastroenterol Hepatol, 2017. **29**(2): p. 231-237.
33. Zhu, X., et al., *Health-related quality of life for gemcitabine and nab-paclitaxel plus radiotherapy versus gemcitabine and S-1 plus radiotherapy in patients with metastatic pancreatic cancer.* Cancer Manag Res, 2018. **10**: p. 4805-4815.
34. Al-Janabi, H., et al., *An investigation of the construct validity of the ICECAP-A capability measure.* Qual Life Res, 2013. **22**(7): p. 1831-40.
35. Keeley, T., et al., *A longitudinal assessment of the responsiveness of the ICECAP-A in a randomised controlled trial of a knee pain intervention.* Quality of Life Research, 2015. **24**(10): p. 2319-2331.
36. Wendel-Vos, G.C., et al., *Reproducibility and relative validity of the short questionnaire to assess health-enhancing physical activity.* J Clin Epidemiol, 2003. **56**(12): p. 1163-9.
37. Yellen, S.B., et al., *Measuring fatigue and other anemia-related symptoms with the Functional Assessment of Cancer Therapy (FACT) measurement system.* J Pain Symptom Manage, 1997. **13**(2): p. 63-74.
38. Goranitis, I., J. Coast, and H. Al-Janabi, *An investigation into the construct validity of the Carer Experience Scale (CES).* Quality of Life Research, 2014. **23**(6): p. 1743-1752.
39. Al-Janabi, H., T.N. Flynn, and J. Coast, *Estimation of a preference-based carer experience scale.* Med Decis Making, 2011. **31**(3): p. 458-68.
40. Mantovani, G., et al., *Cancer-related anorexia/cachexia syndrome and oxidative stress: an innovative approach beyond current treatment.* Cancer Epidemiol Biomarkers Prev, 2004. **13**(10): p. 1651-9.
41. Prado, C.M., et al., *Sarcopenia and cachexia in the era of obesity: clinical and nutritional impact.* Proc Nutr Soc, 2016. **75**(2): p. 188-98.
42. Scott, D., et al., *Health care professionals' experience, understanding and perception of need of advanced cancer patients with cachexia and their families: The benefits of a dedicated clinic.* BMC Palliat Care, 2016. **15**(1): p. 100.
43. Hopkinson, J.B., *The nursing contribution to nutritional care in cancer cachexia.* Proc Nutr Soc, 2015. **74**(4): p. 413-8.

44. Wheelwright, S.J., et al., *A systematic review to establish health-related quality-of-life domains for intervention targets in cancer cachexia.* BMJ Support Palliat Care, 2016. **6**(3): p. 307-14.
45. Malterud, K., V.D. Siersma, and A.D. Guassora, *Sample Size in Qualitative Interview Studies: Guided by Information Power.* Qual Health Res, 2016. **26**(13): p. 1753-1760.
46. Dent, E., et al., *The Cancode interaction analysis system in the oncological setting: reliability and validity of video and audio tape coding.* Patient Educ Couns, 2005. **56**(1): p. 35-44.
47. Vaismoradi, M., H. Turunen, and T. Bondas, *Content analysis and thematic analysis: Implications for conducting a qualitative descriptive study.* Nurs Health Sci, 2013. **15**(3): p. 398-405.
48. Kaushik, V. and C.A. Walsh, *Pragmatism as a Research Paradigm and Its Implications for Social Work Research.* Social Sciences, 2019. **8**(9).
49. Morgan, D.L., *Pragmatism as a Paradigm for Social Research.* Qualitative Inquiry, 2014. **20**(8): p. 1045-1053.
50. M., P., *Variety of qualitative inquiry frameworks: paradigmatic, philosophocal, and theoretical orientations. Qual Res Eval Methods.* 4th ed. ed. 2015.
51. National Cancer Institute. *Common Terminology Criteria for Adverse Events (CTCAE)*. 2018 [cited 2019 12 February]; Available from: https://ctep.cancer.gov/protocoldevelopment/electronic_applications/ctc.htm.
52. Arain, M., et al., *What is a pilot or feasibility study? A review of current practice and editorial policy.* BMC Med Res Methodol, 2010. **10**: p. 67.
53. Sim, J. and M. Lewis, *The size of a pilot study for a clinical trial should be calculated in relation to considerations of precision and efficiency.* J Clin Epidemiol, 2012. **65**(3): p. 301-8.
54. Julious, S.A., *Sample size of 12 per group rule of thumb for a pilot study.* Pharmaceutical Statistics, 2005. **4**(4): p. 287-291.
55. Gelhorn HL, Gries KS, Speck RM, et al. Comprehensive validation of the functional assessment of anorexia/cachexia therapy (FAACT) anorexia/cachexia subscale (A/CS) in lung cancer patients with involuntary weight loss. Quality of life research : an international journal of quality of life aspects of treatment, care and rehabilitation 2019;28(6):1641-1653.

13.0 AMENDMENT LISTING

Amendment Number - 1

Date of amendment – 16th November 2021

Statement of intent –This amendment is in response to initial review by the Human Research Ethics Committee.

List of general changes

A number of very minor spelling and editing errors have been corrected throughout.

The entire protocol has been reviewed for consistency and clarity throughout. Some paragraphs have been moved to a more appropriate section, while other have been removed as duplicate text.

These changes have been tracked but are not specified below.

List of specific changes

Change #1
1.3. Existing evidence of anamorelin for anorexia.
Additional text had been added here to clarify HREC questions.

- Old text
    - The trials failed to meet functional co-primary endpoint but showed that anamorelin was well tolerated and sustainably improved appetite in the majority of participants, a finding not seen in the placebo control arm.
- New text
    - The trials failed to meet functional co-primary endpoint (in this case, hand-grip strength, which was a requirement for approval in Europe and the USA) but showed that anamorelin was well tolerated and sustainably improved appetite in the majority of participants, a finding not seen in the placebo control arm.
    - By contrast, this proposed feasibility study uses a combination of participant-reported measures (appetite) and objective measures directly related to potentially improved health outcomes (improved ability to tolerate and complete anti-cancer therapies on-time and on-dose). These end-points relate directly in the proposed study to outcomes that can deliver improved overall health and wellbeing.

Change #2
1.5. Relevant safety and toxicity information
Additional text had been added here to clarify HREC questions.

- Old text
    - The most common drug-related treatment emergent harms observed were diabetes and hyperglycaemia, occurring in <1% of patients in ROMANA1 and ROMANA 2 trials.

- New text
  - The most common drug-related treatment emergent harms observed were diabetes and hyperglycaemia, occurring in <1% of patients in ROMANA1 and ROMANA 2 trials and more frequent in individuals with per-existing diabetes mellitus.
  - All ECG changes were transient, and none of the QT prolongations were above an absolute value of 450ms or were an increase of >60ms from baseline. Other associated harms were nausea, diarrhea, peripheral oedema and fatigue.

Change #3
3.4.2.1 Inclusion criteria
Inclusion criteria #2 has been clarified.

- Old text
  - Documented histologic or cytologic diagnosis of small cell lung cancer (limited or extensive disease)
- New text
  - Documented histologic or cytologic diagnosis of small cell lung cancer (limited – one lung and/or nearby lymph nodes; or extensive disease – extends beyond single lung, and extended to other lymph nodes or other parts of the body)

Change #4
3.4.2.2 Exclusion criteria
Exclusion criteria #3 has been clarified to exclude those with poorly controlled diabetes

- Old text
  - Diabetes mellitus with secondary organ dysfunction (coronary heart disease, previous stroke, renal insufficiency).
- New text
  - Diabetes mellitus with secondary organ dysfunction (coronary heart disease, previous stroke, renal insufficiency), or poorly controlled diabetes (patients with glycosylated haemoglobin – hbA1c >7% or hyperglycaemia – measured as a fasting blood glucose >7mmol/L or a random blood glucose >11mmol/L) despite receiving clinic-based diabetes care.

Change #5
4.1 Study medication
Table 4-1 has been amended to provide clarification on the precautions and known contraindications

- Old text
  - Interactions with CYP3A4: Co-administration of anamorelin with medicinal products that inhibit (e.g ketoconazole) or induce (e.g. rifampicin) CYP3A4 activity may affect anamorelin plasma concentrations. Concomitant administration of anamorelin with strong CYP3A4 inhibitors should be avoided and the concomitant use of anamorelin with moderate CYP3A4 inhibitors should be done with caution. Concomitant administration of anamorelin with strong CYP3A4 inducers should be done with caution.

- o Cardiac effects: Anamorelin induced prolongation of PR and QRS intervals at supra-therapeutic doses. Therefore, anamorelin should be used with caution in the case of co-administration of medicinal products that may increase its exposure.

- New text
  - o Interactions with CYP3A4: Co-administration of anamorelin with medicinal products that inhibit (e.g ketoconazole, clarithromycin, erythromycin, diltiazem, itraconazole, ritonavir, verapamil) or induce (e.g. rifampicin) CYP3A4 activity may affect anamorelin plasma concentrations. Concomitant administration of anamorelin with strong CYP3A4 inhibitors should be avoided and the concomitant use of anamorelin with moderate CYP3A4 inhibitors should be done with caution. Concomitant administration of anamorelin with strong CYP3A4 inducers should be done with caution.
  - o Cardiac effects: Anamorelin induced prolongation of PR and QRS intervals at supra-therapeutic doses. Therefore, anamorelin should be used with caution in the case of co-administration of medicinal products that may increase its exposure such as antiarrhythmic medications Class I (fast sodium (NA) channel blockers (e.g. quinidine, disopyramide, procainamide, lidocaine, phenytoin, flecainide, propafenone).

Change #6

4.3 Method of assigning participants to treatment groups
Additional text has been added to clarify the 5-items on the FACCT A/CS scale

Old text
  - o Central randomisation will be stratified by limited versus extensive disease and by baseline score of the 5-item Anorexia Symptom Scale Domain from the 12-item FACCT A/CS (≤10 vs >10).

- New text
  - o Central randomisation will be stratified by limited versus extensive disease and by baseline score of the 5-item Anorexia Symptom Scale Domain from the 12-item FACCT A/CS (≤10 vs >10). The 5-item from the FACCT A/CS are as follows: *F1. I have a good appetite; F6. My interest in food drops as soon as I try to eat; F7. I have difficulty eating rich or "heavy" foods; F10. When I eat, I seem to get full quickly; F4. Most food tastes unpleasant to me*.[56]

Change #7

4.7 Drug accountability and destruction
Additional text had been added here to clarify the management of missed doses.

- Old text
  - o Participants will be instructed to bring all unused medication to each study visit during the treatment period for a compliance/accountability check. A participant will be considered to be compliant with investigational medication if she/he takes all study drugs provided.
- New text
  - o Participants will be instructed to bring their diary to each study visit during the treatment period for a compliance/accountability check. A participant will be

considered to be compliant with investigational medication if she/he takes all study drugs provided. If participants have missed any doses, irrespective of the number of doses missed, they will be instructed to not catch up the missed doses, but to continue taking on the next day of treatment. Participants can catchup a missed dose if the next dose is 16 hours away or less.

Change #8
4.8 Cessation of study drug/intervention
The insertion of tub feeding has been added to the list of reasons to cease study drug.

Change #9
5.2.2 Safety assessments
Blood glucose monitoring has been added to the assessments made at each contact

Change #10
6.1 Laboratory measures
A new paragraph has been added to describe the process for blood glucose monitoring
New text

- ***Blood Glucose Monitoring***
  All participants with diabetes enrolled in this study will be required to monitor their blood glucose levels in consultation with their treating physician. People who are insulin dependent will be encouraged to commence daily monitoring (if not already doing so), and those controlled with lifestyle measures and oral medication, blood glucose and HbA1c concentrations will be monitored at specified study visits and at any other healthcare interaction, along with participant review of their diet control. Changes in blood glucose levels will be reviewed as adverse events.

Change #11
6.2 Clinical and physical measures
Additional text has been inserted to clarify cardiac assessment

- New text
  - The medical examination will also include a complete cardiac history and examination to assess for previous or recent cardiac events, and will make use of physical examination, close review of the medical record, and assessment of the New York Heart Association Classification, if appropriate.

Change #12
Table 6-1 Schedule of study assessments
Additional columns have been inserted to describe the measures to be assessed at Exit or early termination, and during follow-up.

Change #13
7.2 Visit 2
Text has been changed to clarify that the study medication will be delivered to the participant home.

- Old text

- o The patient will be randomised and study kit be dispensed. Study drug should be taken next day after Visit 2 (at least 1 hour prior to first meal of the day).
- New text
  - The patient will be randomised with the study kit to be delivered to their home later that day. Study drug should be commenced the next day after Visit 2 (at least 1 hour prior to first meal of the day).

Change #14
8.8 Treatment commencement
Text has been added to clarify first dosing.

- New text
  - The first dose should be taken the following morning (prior to the first meal) after provision of the blinded medication, this is day 1. Dosing is thereafter to continue each morning.

Change #15
9.1.3 Medical Monitor
Text has been added to describe the safety overview processes of the study using a medical monitor.

- Old text
  - o An independent DSMC will be contracted. The primary role of the DSMC will be to monitor AEs and SAEs. The committee will have access to coded safety reports which will be reviewed at six (6) monthly intervals, or as agreed by the DSMC. In addition, any emerging safety issues will be reviewed by the DSMC on an ad hoc basis if required. Specifically, the DSMC will receive SAEs as part of the established reporting mechanism (email notification of the report from the ITCC within 24 hours) if the event is unexpected and related to the study intervention. In addition, the DSMC will receive a summary report of all AEs, these will be discussed as a standing agenda item, with the discussions, actions and outcomes recorded. The DSMC will also receive an updated literature summary at each meeting, which will address new published literature that may have an impact on the study.
- New text
  - A Medical Monitor (MM) will be assigned to review and evaluate information relevant to the safety of the investigational product used. The MM has the responsibility to review and evaluate information relevant to the product safety throughout the implementation of the protocol at all participating sites.
    The MM will be responsible for providing safety oversight and reviewing the protocol (e.g., study halting rules) and information about the study product as it becomes available, such as the reportable safety events (SAE's). The MM, in consultation with the protocol investigator team and the Sponsor will provide safety review during the execution of the clinical trial. This oversight includes reviewing safety information and providing applicable recommendations. The MM will provide recommendations, as appropriate, to members of the study and investigator team. This data and safety review facilitates early detection of safety signals and maximizes the chances for continued appropriateness of the research and protection of human participants.

Based on a synthesis of this information, the MM will provide appropriate recommendations to the Sponsor, participating recruiting sites and approving HRECS.

Change #16
9.1.7 Criteria for assessing causality
Text has been added to clarify assessing causality of adverse events.

- New text
  - As this study is blinded, all adverse and serious adverse events should be assessed for causal relationship assuming that the participant has been receiving anamorelin. When determining whether an AE or SAE is expected or not, please refer to the reference safety information provided in the Investigator Brochure. If the event is listed in the Investigator Brochure or specified as a symptom of special interest in section 9.1.9, the AE or SAE should be assumed to be 'Possible', 'Probable' or 'Definite' unless there is a clear alternative cause.

Change #17
9.1.9 NCI CTC AE
The list of symptoms of interest has been expanded to enable symptoms to be separately graded in accordance with standard NIC CTC AE grading.

- New text
  - Symptoms will be identified during each visit using criteria established by the National Cancer Institute, and will be graded accordingly. Specifically, for this study, the symptoms of interest will be:
    - Symptomatic hyperglycaemia (dizziness, cold sweat, loss of consciousness, abdominal pain, shortness of breath, confusion), constipation, nausea, vomiting.
    - Blood glucose increased (investigation)
    - Symptoms of cardiac dysfunction (PR prolongation and increased QT interval measured on ECG), blurred vision, breathlessness, chest pain, syncope, palpitations
    - Headache
    - Peripheral oedema

Change #18
9.4.2 Direct access to source data
As a feasibility study data will not be shared. We have amended this section to incorporate the wording from HREA application. We have also incorporated the same wording into the PICF.

- Old text
  - In addition de-identified data will be made available for meta-analysis and where requested by journals for publication purposes.

- New text
  - Increasingly, there is a requirement to make the data available at publication dependent on the journal requirements and this would be done in a summarised

and de-identified format. So de-identified data may be made available to bona fide researchers for meata-nalysis and where requested by journals for publication purposes. Regulatory authorities may require use of the data for subsidy applications. In addition, the aggregated study data will be used to inform the next phase of the study. Any future use of the study data would be at the discretion of the Sponsor/data custodian.

Change #19
11.3.5 Risks associated with study procedures – DEXA scans
New text has been added to fully explain any potential risks associated with DEXA scans. This has also been added to the PICF

- New text
    - As part of this study, participants will be required to undergo DEXA scans at Visit 2, Visit 5 or Visit 7 or withdrawal to track changes in weight and associated changes in fat and muscle mass. Whilst the scans only take 25 minutes to complete, this is additional imaging and radiation exposure than would be typically required as part of routine standard of care. However, the DEXA scan is very safe for most people as it uses very low level of x-ray energy. As part of everyday living, everyone is exposed to naturally occurring background radiation and receives a dose of about 2 millisievert (mSv) each year. The effective radiation dose from the extra DEXA scans required as part of this study is estimated to be less than 0.02 mSv. At this dose level, no harmful effects of radiation have been demonstrated as any effect is too small to measure. The risk is believed to be minimal. Participants will be advised of the minimal risks associated with the DEXA scans in the PICF.

Amendment Number - 2

Date of amendment – 27 January 2022

Change #1
The contracted pharmacy, The Pharmacy Common, has been replaced with another licenced community pharmacy. All reference to The Pharmacy Common, or TPC, has been replaced with the third party pharmacy.

Change #2
11.3.5 Risks associated with study procedures – DEXA scans
We have updated the information in this section to include the maximum amount of radiation dose provided in the reports from all site where this study will be conducted (changed from 0.02mSv to 0.07mSv) which has also been included in the PICF.

- Old text
  - The effective radiation dose from the extra DEXA scans required as part of this study is estimated to be less than 0.02 mSv.

- New text
  - The effective radiation dose from the extra DEXA scans required as part of this study is estimated to be less than 0.07 mSv.

Change #3
As part of the cytokine biomarkers, only IL-6 will be assessed. This will be measured at baseline, Week 4 and at participant's last visit (i.e. either at Week 12 if not participating in the extension phase, or Week 24 if opting into the extension phase, or at the time of withdrawal). We have removed IL-1 alpha from the assessments.

Change #4
6.7 Patient Reported Symptom Measure
Participant acceptability of the study is a primary endpoint of this study, under desirability. A paragraph detailing the tool that will be used to assess participant acceptability of the study and proxy response measurement has been added to the protocol.

Amendment Number - 3

Date of amendment – 7 April 2022

Change #1
•Study title – We have included an acronym for the study which is LUANA trial (LU- lung cancer; AN- anamorelin; A- anorexia)

Change #2
•Sub-study – Information concerning the sub-study has been removed from the main protocol and will be detailed in a separate document – the Sub-study Procedures Manual. To this effect, PICF for the main trial have also been amended and new PICF for the sub-study (including PICFs for clinicians, participants and caregivers) are provided as separated documents.

Change #3
•Objectives/ assessments – We have moved the primary efficacy parameters to its correct place. We have also expanded details in secondary assessments section to add information of what it will entail.

Change #4
•Study procedures – Given the nature and progression of SCLC, we acknowledge that planned treatment may start immediately after diagnosis so in order to allow time for screening and baseline assessments to be undertaken, we have extended the time between Visit 1 and Visit 2 and Day1 of study medication. In the previous protocol version (1.5), there were 7 days between screening and baseline which could also be conducted at the same day. Current change allows for baseline to be undertaken at any time after screening and prior to day 1 of Cycle 2 of scheduled chemotherapy. If chemotherapy is not planned, Day 1 should occur within three weeks of screening visit. The study diagram (section 3.2) has also been updated to reflect these changes.

- Old text
  - "Given the urgency of cancer treatment in SCLC, and as to minimise the impact of the study on patients, as well as to maximise engagement with the study, screening and baseline assessments for the study can be conducted on the same day, and as close to starting the study as possible (preferably, if the person can commence the study within 1-2 days). Study treatment will start within one week after randomisation."

- New text
  - "Participants will be recruited using advertisement in waiting rooms and clinician referrals. Adults (aged ≥18 years) with a confirmed diagnosis of SCLC are referred to the study nurse at each participating centre and assessed for eligibility. People who meet all of the inclusion criteria and none of the exclusion criteria are eligible to enter this study. All participants will be required to sign an informed consent prior to performing any study

procedures. Screening assessments will be undertaken to confirm eligibility. Randomisation will occur after eligibility is confirmed and baseline assessments are collected" …. "Treatment (Day 1) will initiate as soon as practicable; ideally prior to cycle 1 of chemotherapy and no later than one day prior to scheduled date of cycle 2 of planned chemotherapy. If chemotherapy is not planned, Day 1 should occur within three weeks of screening visit."

Change #5

•Inclusion/Exclusion – We have emphasised in bold text that "**under no circumstances should the study delay the routine treatment for SCLC".** Further, we have explained in exclusion criteria that if participants are taking some of listed medications for short-term and as part of routine chemotherapy/radiation therapy standard protocol, this will not exclude them from the study. The medications include androgenic compounds such as corticosteroids, antiemetics such as olanzapine and metoclopramide and others.

Furthermore, a note has been added to inform what the term "planned therapy" in inclusion criteria means: i.e. P*lanned therapy includes the period from having a defined treatment schedule and study medication starting up to one day prior to day one of Cycle 2 of chemotherapy.*

In Withdrawal criteria we had listed categories that are part of cessation of medication and not linked with withdrawal so we have amended this part and moved the relevant information to section 4.8. Similarly, we have moved text from 8.11 withdrawal assessments to section 8.10 (end of treatment assessments)

The new text in section 3.4.3 reads as follows: "*Participants may withdraw their consent and discontinue the study treatment at any time. They may withdraw their consent for continued administration of the study medication with or without withdrawing consent from all protocol-specified assessments, follow-up procedures and for the study team to use already collected data. Every effort should be made to encourage participants to return for scheduled visits to undergo study procedures even if they discontinue the study drug. If participants withdraw study participation at any time, they will be asked if they can participate in one final clinical visit and whether they would participate in a follow-up telephone call seven days later."*

8.10 – *"If early cessation due to an AE, all associated documents will be completed (Serious Adverse Event (SAE) report, AE assessment score etc.). An AE with a grade of 3 (that has not responded to symptomatic treatment instituted by the treating physician according to local protocols) or 4 will activate treatment cessation and an AE report will need to be completed and submitted. Please refer to Section 10 for further information about AE and SAE reports."*

Change #6
•4.2 Dosing schedule and 8.8 Treatment Commencement – These sections have been amended for clarity and for additional information to reflect the changes to the timeline between screening and Day 1 of study medication.

4.2 - *Participants will take the first dose of intervention on Day 1, which is the day after receipt of the medication. If a fasting state is not possible on Day 1, participants will start study drug on the next possible day and daily thereafter until Week 12 or up to week 24, if a participant opts in for blinded continuation in the extension phase. If participants have missed any doses, irrespective of the number of doses missed, they will be instructed to not catch up the missed doses if the missed dose was more than 8 hours earlier, but to continue taking on the next day of treatment. Participants will be supplied with study drug within business days after randomisation (i.e. up to one day prior to starting Cycle 2 of chemotherapy) and at Week 18.*

8.8 - *The study medication will commence on the same day of delivery if participant has not eaten first meal of the day (start Day 1). If a fasting state is not possible on Day 1, participants will start study drug on the next possible day but no later than one day prior to planned day 1 of Cycle 2 of chemotherapy. Dosing is thereafter to continue daily, 1 hour prior to the first meal of the day.*

Change #7
•Editorial changes – we have simplified wording/ edited / provided further details or examples throughout the protocol to improve clarity. We have also in some instances, included text into tables to provide structure for detailed information (such as the ones included in section 7.0 participant timeline) or simply moved text around (or deleted repetitive statements) to avoid repetition and to make sure it goes under the most appropriate heading. Some examples of changes are listed below:

- The term "Week 1" has been replaced by "baseline visit"
- We reinforced that the multi-modal intervention will be delivered in the same way: *At baseline, all participants will receive standardised and structured physical activity and diet advice leaflets from the study nurse as part of a multi-modal intervention. All participants will receive the same information delivered in the same way.*
- The information about missed doses was confusing with the 16 hours so we edited it for clarity: *If participants have missed any doses, irrespective of the number of doses missed, they will be instructed to not catch up the missed doses if the missed dose was more than 8 hours earlier, but to continue taking on the next day of treatment.*
- Information on study assessment and meaning of 28 days: "*The study period will include 28 days after medication cessation (4 weekly follow-up period) or until death (whichever timeframe is shorter).*"
- The term "DSMC" has been replaced by "Medical Monitor" or "MM"

Change #8

•Randomisation changed from REDCap to Central Randomisation Service: It has been found that the proposed randomisation via REDCap does not have an adequate audit trail. The procedure for randomisation has been changed to be conducted via a Central Randomisation Service using telephone and email communication. Relevant changes to the text are detailed below and in sections 4.3 and 8.6 in the protocol.

4.3 Method of assigning participants to treatment groups – *"On notification of a participant, the delegated pharmacist at a third party pharmacy will email the ITCC Central Randomisation Service (Randomisation@uts.edu.au) providing the required registration details and receive the treatment allocation via reply email. This allocation will be printed or saved electronically into the pharmacy records. The participant ID, allocation code, dates of request, preparation, and dispensing will be recorded in a log maintained by the pharmacist and supplied to the central registry on each randomisation."*

8.6 Procedure to request randomisation – *"The randomisation request will take the form of the prescription of the study medications. Upon receipt of the prescription, the pharmacist at the third party pharmacy will then prepare the study medication according to the allocation received from the ITCC Central Randomisation Service. This allocation email will be filed in the pharmacy study folder."*

Change #9

•We have added in the protocol more detailed information about the blinding/ unblinding procedures for clarity:

4.4 - "*Treatment allocation will not be disclosed to participants, study staff, treating clinicians or investigators. The code will only be broken in cases of extreme emergency (refer to 4.5). The drug manufacturer will ensure study drug and placebo appear identical in regards to appearance, smell and taste. All medication will then be secondary packaged and dispensed in blinded packaging on a per participant basis by a contracted third party pharmacy, according to the treatment allocation derived from the randomisation schedule (refer to 4.6).*"

4.5 – "The code will only be broken in cases of extreme emergency. Such situations only include where knowledge of the allocation will have consequences for clinical decision making in consultation with the Lead Investigator who will have access to the sealed unblinding schedule. The Lead Investigator, therefore, must be contacted in the first place. Clinical staff will be able to discuss the clinical situation with the Lead Investigator to determine the urgency and need for unblinding and will be informed by the Lead Investigator of the treatment allocation based on these discussions."

Change #10

•4.6 Drug supply– Detailed information on drug supply and external pharmacy were included in the protocol. In addition, the number of tablets/kit have been updated to allow for variances in days until study Visit 5 when participants will decide if they want to participate into the extension phase.
"The drug manufacturer will provide the bulk tablets (active and matching placebo). A licensed local pharmaceutical development and manufacturing organisation contracted by the Sponsor will import and store the bulk supply. This will be made available on a Just-in-Time basis to their sub-contracted central pharmacy (referred to as third party pharmacy) for the dispensing activity. The third party pharmacy will secondary package the bulk tablets into study medication kits (90 tablets/kit for the first 12 weeks of treatment period and 80 tablets/kit for the extension phase). The third party pharmacy, will dispense the study medication on a per participant basis upon receipt of a valid prescription. A log will be maintained of the kit dispensed to each participant.

Change #11
•4.7 Drug accountability and destruction – Given participants will receive medication at home and will be asked to record daily if tablet was taken or not (and reason), compliance will involve checking participant's records. In the instances when participant is not recording this information daily, they will be asked to bring any unused medication at study exit so that the study nurse can count remaining tables. The new text reads as follows:
"*Participants will be instructed to bring their diary to each study visit during the treatment period for a compliance/accountability check. Compliance will be assessed at visits at weeks 4, 8 and 12 (plus 18 and 24 for those in the extension phase) using participant record of self-administration (i.e. participant diary) OR counting of the remaining tablets at treatment cessation, if the information has not been recorded in the participant diary. A participant will be considered to be compliant with investigational medication if she/he takes all study drugs provided."*

Furthermore, participants will receive specific details on how to return unused medication to the pharmacy, therefore, we have amended the text to reflect this:

"After the last dose is taken and study medication is ceased, participants will return all unused study medication to the contracted licensed pharmaceutical development and manufacturing organisation using the return-addressed satchel provided to the patient with the dispensed drug. Upon receipt of the unused study medication, final accountability will be completed and documented in the study pharmacy records.

All unused study medication will be destroyed in a manner consistent with the applicable regulations governing destruction in each state or country. The contracted licensed pharmaceutical development and manufacturing organisation Standard Operating Procedures and state regulations are to be referred to and adhered to at all times."

Change #12

•4.8 Cessation of study drug/intervention – We have edited this section for clarity, including part of text that was under withdrawal criteria as mentioned previously. We have also included information about planned follow-up visits.

The text now reads: *If a participant prematurely discontinues treatment with anamorelin/placebo at any time prior to completing the 12-week visit, the Investigator must try his/her best to contact the person for scheduled visits…. If participants discontinue study participation at any time, they will be asked if they can participate in one final clinical visit and whether they would participate in a follow-up telephone call seven days later.*

Change #13

•6.1 Laboratory measures – Ensuring that the study involves minimal burden to participants is crucial, therefore, in some instances (e.g., if clinical picture has not otherwise changed), blood samples taken as part of diagnosis as well as pre-chemo/radiation therapy cycles can be used for screening, baseline and follow up visits. This information has been added in the protocol in replacement to information about blood works being accepted if done in the preceding 2 weeks. Further, urinalysis will not be done as part of this study anymore and this has been amended throughout the protocol and PICF.

The whole section that details blood chemistry has been updated for clarity. Manly by moving text around and expanding acronyms. The text under blood glucose monitoring has also been edited to add details about when the assessments will be done: "*All participants with diabetes enrolled in this study will be required to monitor their blood glucose levels in consultation with their treating physician. People who are insulin dependent will be encouraged to commence daily monitoring (if not already doing so) in consultation with their treating physician. During their participation in the study, blood glucose levels and HbA1c concentrations will be assessed as part of blood chemistry at specified study visits [including, screening visit (Visit 1), Week 4 (Visit 3), Week 12 (Visit 5) and at Week 24 (Visit 7, if applicable)]. Changes in blood glucose levels will be reviewed as adverse events.*"

Regarding the urine pregnancy test prior to first dose, this has been amended as follows: "*A negative urine pregnancy test will be needed at baseline prior to first dose of investigational product.*" Therefore, participants will not need to repeat a pregnancy test 24h prior to taking the medication given they will have done a urine pregnancy test at baseline.

Change #14

•Clinical and physical measurements/ clinical diagnosis – Again, for clarity, some minor edits on the text have been done. We have highlighted that new CT scans will not be asked as part of this trial and only those done clinically as part of usual care will be reviewed for study purpose. We have expanded details of what parts of the body will be examined as part of the dedicated physical exams for clarity. We have also included further details of what information will be collected as part of main clinical diagnosis.

Change #15
•ECG – Given ECG will be done as part of screening, we will not repeat it at baseline. This information has been amended throughout the protocol and PICF.

Change #16
•Caregiver assessments - We have mistakenly included in previous protocol version, ICECAP-A assessment for caregivers, this will not be done and has been amended. The information about EuroQol was missing in this section so we have included it.

Change #17
•Tables 6-1 and 6-2 with schedule of study assessments have been amended for clarity and to accommodate the above mentioned changes. Similarly, Study visit schedule diagram (section 7.8) has been modified.

Change #18
•Section 7.0 participant timeline - as mentioned previously, this section has been extensively edited to include the assessments/text into tables to provide structure for detailed information. We have also made it easier within tables for researchers to identify what assessments are part of clinician-based assessments and which ones are patient reported outcomes. Furthermore, the tables make it visually easier to identify assessments for patients vs caregivers.

Change #19
•Section 8.1 We have added the following text to referral section: *"All people with a confirmed diagnosis of small cell lung cancer should be referred to the study as soon as possible after diagnosis to enable eligibility screening and baseline assessments to be conducted prior to Cycle 2 of chemotherapy or within three weeks from screening visit, if there is no planned chemotherapy. Therefore,…"*

Change #20
•Section 8.3 – We have added to this section for reinforcement of eligibility, that a primary caregiver should be 18 years and over and is defined as a spouse/relative, partner or friend of the patient who provides care for the person and not someone who has been employed to care for the person.
