# Supplementary material for: Phase II, double blind, placebo controlled, multi-site study to evaluate the safety, feasibility and desirability of conducting a phase III study of anamorelin for anorexia in people with small cell lung cancer: A study protocol (LUANA trial)
Source: PLoS One. 2023 May 17;18(5):e0285850. doi: 10.1371/journal.pone.0285850 (PMC10191352; doi:10.1371/journal.pone.0285850)
Supplement: Attachment — Submitted filename: Mariana S et al. PLOSONE.pdf [file pone.0285850.s003.pdf]

Mariana S. Sousa et al. Presented a Study Protocol LUANA trial, a randomized phase 2 trial with anamorelin for anorexia in people with small cell lung cancer.

My main concern is about the novelty of this trial. I would like to mentioned the fact that a previous trial using the design and the same drug is alrealdy ongoing in NSCLC: "NCT03743064 A Phase 3, randomized, double-blind, placebo-controlled, multicenter study to evaluate the efficacy and safety of anamorelin HCl for the treatment of malignancy associated weight loss and anorexia in adult patients with advanced non-small cell lung cancer (NSCLC)." So, the evaluation of the the safety, feasibility and desirability in LUANA trial sounds futile to me. The drugs used in SCLC with platinum-based chemotherapy associated with immunotherapy is also the backbone of NSCLC treatment . Therefore, ANAM 17-21 trail in NSCLC does already address LUANA phase II design presented in this work.

I am also concern about the fact the list of exclusion criteria:" Pleural effusion requiring thoracentesis. Pericardial effusion requiring drainage. Oedema requiring regular diuretics. Ascites requiring drainage. Oesophagitis, gastritis, Cognitive impairment…." Will exclude most of patient treated in daily routine with advanced SCLC which is a symptomatic disease related to high proportion of metastasis. Only "super Fit" patient with SLCLC and cachexia will be enrolled in the trial. I am quite sure that this kind of patient are highly selected population, consequently the accrual in the trial as well as the clinical application in daily routine practice is questionable.

Futhermore, this kind of trial should be a pantumor trial, unless if there is strong rational to support a specific tumor histology subtype or specific oncogenic driver.
